# Crystal structures of four gold(I) complexes [Au*L*_2_]^+^[Au*X*_2_]^−^ and a by-product (*L*·*L*H^+^)[AuBr_2_]^−^ (*L* = substituted pyridine, *X* = Cl or Br)

**DOI:** 10.1107/S2056989024005437

**Published:** 2024-06-18

**Authors:** Cindy Döring, Peter G. Jones

**Affiliations:** a Institut für Anorganische und Analytische Chemie, Technische Universität Braunschweig, Hagenring 30, D-38106 Braunschweig, Germany; Universität Greifswald, Germany

**Keywords:** crystal structure, gold, pyridine, halide ligands, aurophilic contacts

## Abstract

Bis(2-methyl­pyridine)­gold(I) di­bromido­aurate(I), [Au(C_6_H_7_N)_2_][AuBr_2_], (**1**), crystallizes in space group *C*2/*c* with *Z* = 4. Both gold atoms lie on twofold axes and are connected by an aurophilic contact. A second aurophilic contact leads to infinite chains of alternating cations and anions parallel to the *b* axis, and the residues are further connected by a short H⋯Au contact and a borderline Br⋯Br contact. Bis(3-methyl­pyridine)­gold(I) di­bromido­aurate(I), [Au(C_6_H_7_N)_2_][AuBr_2_], (**2**), crystallizes in space group *C*2/*m* with *Z* = 2. Both gold atoms lie on special positions with symmetry 2/*m* and are connected by an aurophilic contact; all other atoms except for one methyl hydrogen lie in mirror planes. The extended structure is closely analogous to that of **1**, although the structures are formally not isotypic. Bis(3,5-di­methyl­pyridine)­gold(I) di­chlor­ido­aurate(I), [Au(C_7_H_9_N)_2_][AuCl_2_], (**3**) crystallizes in space group *P* with *Z* = 2. The cation lies on a general position, and there are two independent anions in which the gold atoms lie on inversion centres. The cation and one anion associate *via* three short H⋯Cl contacts to form a ribbon structure parallel to the *b* axis; aurophilic contacts link adjacent ribbons. Bis(3,5-di­methyl­pyridine)­gold(I) di­bromido­aurate(I), [Au(C_7_H_9_N)_2_][AuBr_2_], (**4**) is isotypic to **3**. Attempts to make similar compounds involving 2-bromo­pyridine led instead to 2-bromopyridinium di­bromido­aurate(I)–2-bromo­pyridine (1/1), (C_5_H_5_BrN)[AuBr_2_]·C_5_H_4_BrN, (**5**), which crystallizes in space group *P* with *Z* = 2; all atoms lie on general positions. The 2-bromo­pyridinium cation is linked to the 2-bromo­pyridine mol­ecule by an N—H⋯N hydrogen bond. Two formula units aggregate to form inversion-symmetric dimers involving Br⋯Br, Au⋯Br and H⋯Br contacts.

## Abstract 2

In the four main structures, the anions and cations are connected by aurophilic contacts, hydrogen bonds C—H⋯halogen and (in two cases) C—H⋯Au contacts. In the by-product without pyridinic N coordination a N—H⋯N bond dominates the packing pattern supported by Br⋯Br, H⋯Br, and Au⋯Br contacts.

## 1. Chemical context

The first X-ray structural results on pyridine complexes of gold(I) were reported by the group of Strähle (Adams *et al.*, 1982 ▸), one of the pioneers of structural gold chemistry, who established that the compounds with stoichiometry (py)Au*X* (py = pyridine, *X* = Cl and I) were in fact ionic, [Au(py)_2_]^+^[Au*X*_2_]^−^. In both compounds, the ions were linked by short Au⋯Au contacts to form tetra­nuclear chains anion⋯cation⋯cation⋯anion, with a linear sequence Au⋯Au⋯Au⋯Au for *X* = I but a zigzag for *X* = Cl. For *X* = I, the Au⋯Au distances were shorter (peripheral 2.990, central 3.291 Å) than for *X* = Cl (3.249, 3.416 Å). Such contacts have now proved to be quite common for Au^I^ centres and have been intensively studied, in particular by Schmidbaur, who termed them ‘aurophilic contacts’ (see *e.g.* Schmidbaur & Schier, 2008 ▸, 2012 ▸). We recently redetermined the structure of the iodine derivative, using the improved methods now available, as a student project and obtained Au⋯Au distances of 2.9784 (3) and 3.2575 (5) Å (Döring *et al.*, 2018 ▸).
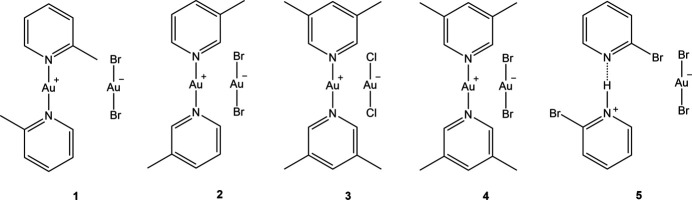


Our series of publications ‘Gold complexes with amine ligands’ consists of sixteen numbered publications (and several, mostly earlier, publications that were not numbered). Parts 12–15, published recently (Döring & Jones, 2023*a* ▸,*b* ▸, 2024*a* ▸,*b* ▸), involved complexes of cyclic secondary amines. We have employed the term ‘amine’ liberally to include aza-aromatics, mostly pyridine or substituted pyridines. Some time ago, we investigated complexes of substituted pyridines with gold(I) halides and reported the structures of the following compounds: chlorido­(2-methyl­pyridine)­gold(I), a mol­ecular complex that forms an almost linear chain polymer *via* Au⋯Au contacts of 3.1960 (4) Å; bis­(3-methyl­pyridine)­gold(I) di­chlorido­aurate(I), which also forms a chain polymer, in which alternating anions and cations are linked by Au⋯Au contacts of 3.1538 (12) Å, with Au⋯Au⋯Au angles of 180° and 158.25° at the gold atoms of the cations and anions, respectively (Jones & Ahrens, 1998 ▸); bis­(3-bromo­pyridine)­gold(I) di­chlorido­aurate(I), which forms zigzag tetra­nuclear units of the form anion⋯cation⋯cation⋯anion, with Au⋯Au contacts of 3.2681 (7) and 3.3113 (10) Å (Freytag & Jones, 2000 ▸); and the isotypic compounds bis­(4-methyl­pyridine)­gold(I) di­chlorido­aurate(I) and bis­(4-methyl­pyridine)­gold(I) di­bromido­aurate(I), which form linear trinuclear aggregates anion⋯cation⋯anion with Au⋯Au contacts of 3.1874 (2) or 3.1796 (2) Å, respectively (the second cation forms no aurophilic contacts) (Döring & Jones, 2013*a* ▸). The structure of bis­(4-methyl­pyridine)­gold(I) di­chlorido­aurate(I) had previously been reported by Lin *et al.* (2008 ▸) but was redetermined to resolve a space group problem. It is noteworthy that the ionic complexes [*L*_2_Au][Au*X*_2_] (*L* = pyridine ligand, *X* = halogen) are commoner than the mol­ecular *L*Au*X* (see also Section 4). We have found corresponding derivatives with pseudohalogens to be exclusively ionic for thio­cyanates (Döring & Jones, 2014 ▸ and Strey *et al.*, 2018 ▸), whereas cyanides were exclusively mol­ecular (Döring & Jones, 2013*b* ▸). One of us (PGJ) was also peripherally involved in research on organometallic complexes of gold, several of which contained pyridine ligands; this research centred on the groups of Laguna (Zaragoza) and Vicente (Murcia), see *e.g.* Barranco *et al.* (2004 ▸) and Vicente *et al.* (1998 ▸).

We have now returned to complexes involving pyridine ligands. In this publication we describe the structures of four gold(I) halide derivatives of empirical formula *L*Au*X*, all of which proved to be ionic compounds of the form [Au*L*_2_]^+^[Au*X*_2_]^−^ (*L* = substituted pyridine, *X* = Cl or Br), together with one by-product. The next publication (in preparation) will describe complexes of the form *L*Au*X*_3_ for the same ligand type.

The reader should note that the trivial names picoline (= methyl­pyridine) and lutidine (= di­methyl­pyridine) have often been used (also by us) in the literature.

## 2. Structural commentary

We note at the outset that, for compounds consisting of more than one residue, it is to some extent arbitrary which aspects belong to the *Structural commentary* and which to the *Supra­molecular features* (next section). In this section we describe only structural aspects within the asymmetric unit, extended where necessary to generate complete ions.

**Figure 1:**
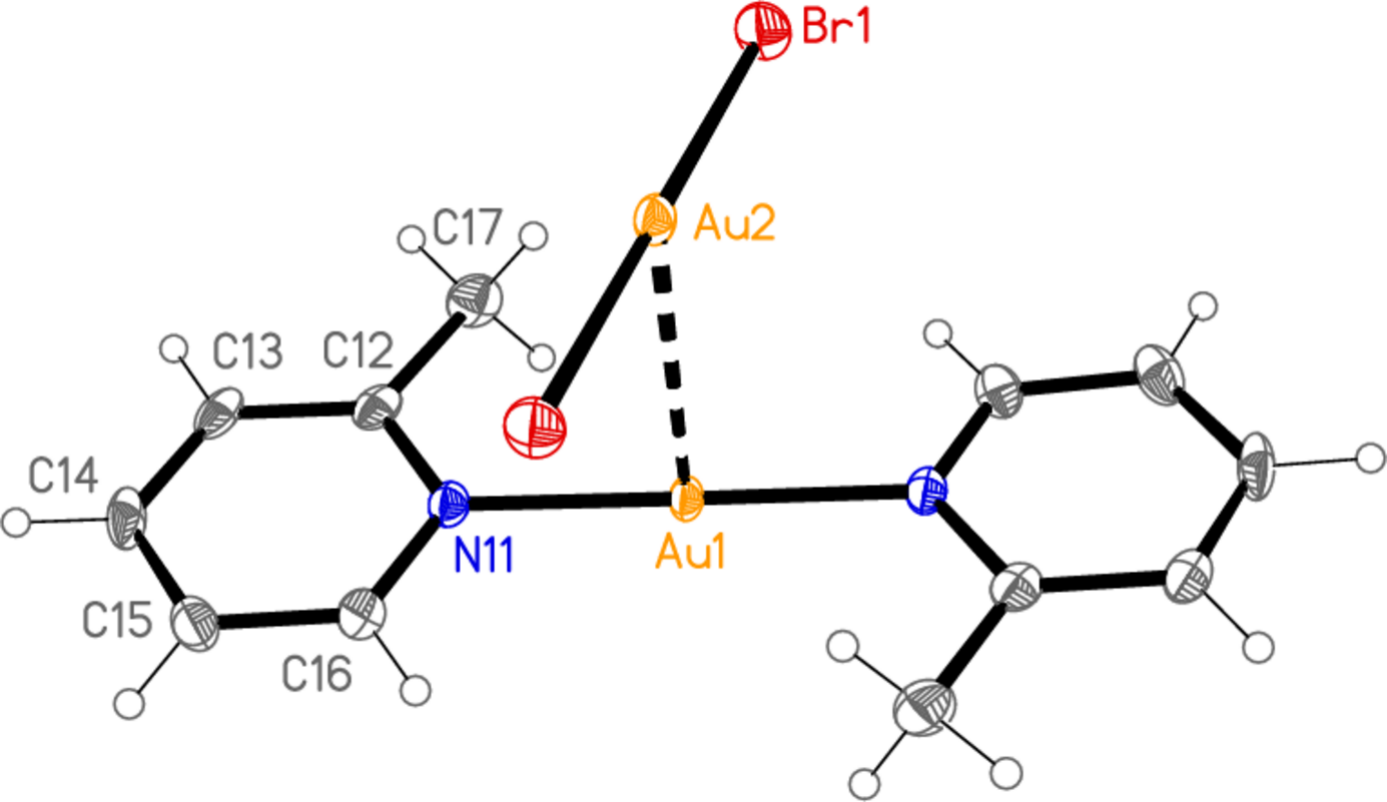
The structure of compound **1** in the crystal, showing the asymmetric unit (labelled) extended by symmetry. The dashed line represents an aurophilic attraction. Ellipsoids correspond to 50% probability levels.

The structure of bis­(2-methyl­pyridine)­gold(I) di­bromido­aurate(I) (**1**), which crystallizes in space group *C*2/*c* with *Z* = 4, is shown in Fig. 1 ▸, with selected dimensions in Table 1 ▸. The corresponding chlorido derivative (Jones & Ahrens, 1998 ▸) is mol­ecular rather than ionic; it is not clear which factors determine the ionic or mol­ecular nature of compounds with stoichiometry *L*Au*X*, and we did not attempt to find alternative forms of the compounds described here (*e.g*. by carrying out extensive recrystallization experiments). Both gold atoms lie on the twofold axis (0, *y*, 0.75) and are connected by an aurophilic contact of 3.1904 (4) Å. The coordination axes N11—Au1—N11′ and Br1—Au2—Br1′ are approximately perpendicular to each other across the Au⋯Au contact (see torsion angles in Table 1 ▸). The inter­planar angle of the two rings is 4.31 (2)°, with the methyl groups on opposite sides of the rings.

**Figure 2:**
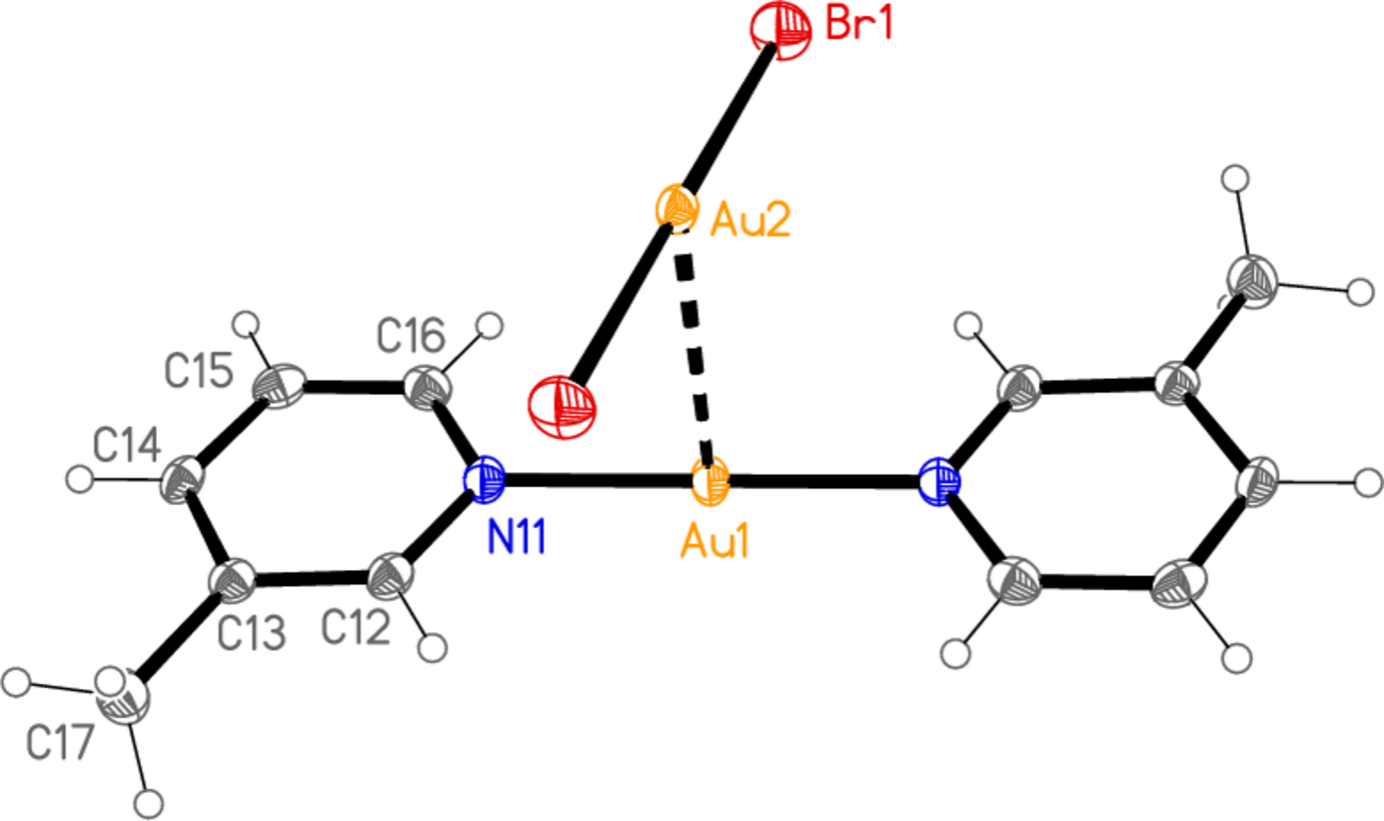
The structure of compound **2** in the crystal, showing the asymmetric unit (labelled) extended by symmetry. The dashed line represents an aurophilic attraction. Ellipsoids correspond to 50% probability levels.

The structure of bis­(3-methyl­pyridine)­gold(I) di­bromido­aurate(I) (**2**), which crystallizes in space group *C*2/*m* with *Z* = 2, is shown in Fig. 2 ▸, with selected dimensions in Table 2 ▸. It is not isotypic to the chlorido derivative (Jones & Ahrens, 1998 ▸; see next section). Both gold atoms of **2** lie on special positions with symmetry 2/*m*, and all other atoms except for one methyl hydrogen (see *Refinement*) in mirror planes at *y* = 0 or 0.5. The gold atoms are connected by an aurophilic contact of 3.22048 (6) Å. Again, the coordination axes at the gold atoms are approximately perpendicular to each other (see torsion angles in Table 2 ▸). The crystallographic symmetry means that the coordination at both gold atoms is exactly linear, the rings are exactly coplanar, and the coordination axes are exactly perpendicular to the Au⋯Au contacts while roughly perpendicular to each other.

**Figure 3:**
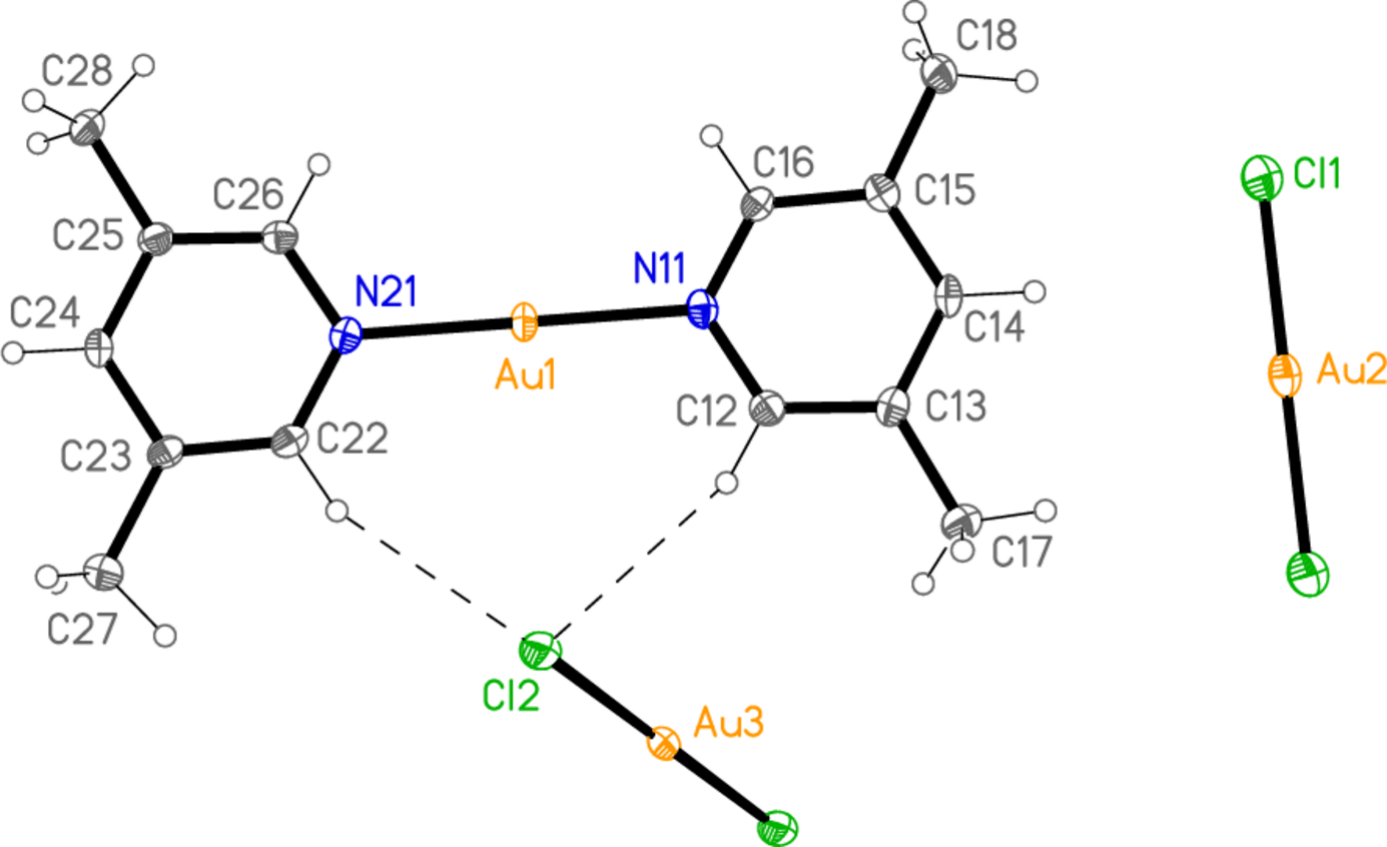
The structure of compound **3** in the crystal, showing the asymmetric unit (labelled) extended by symmetry. Ellipsoids correspond to 50% probability levels. Dashed lines indicate short H⋯Cl contacts.

**Figure 4:**
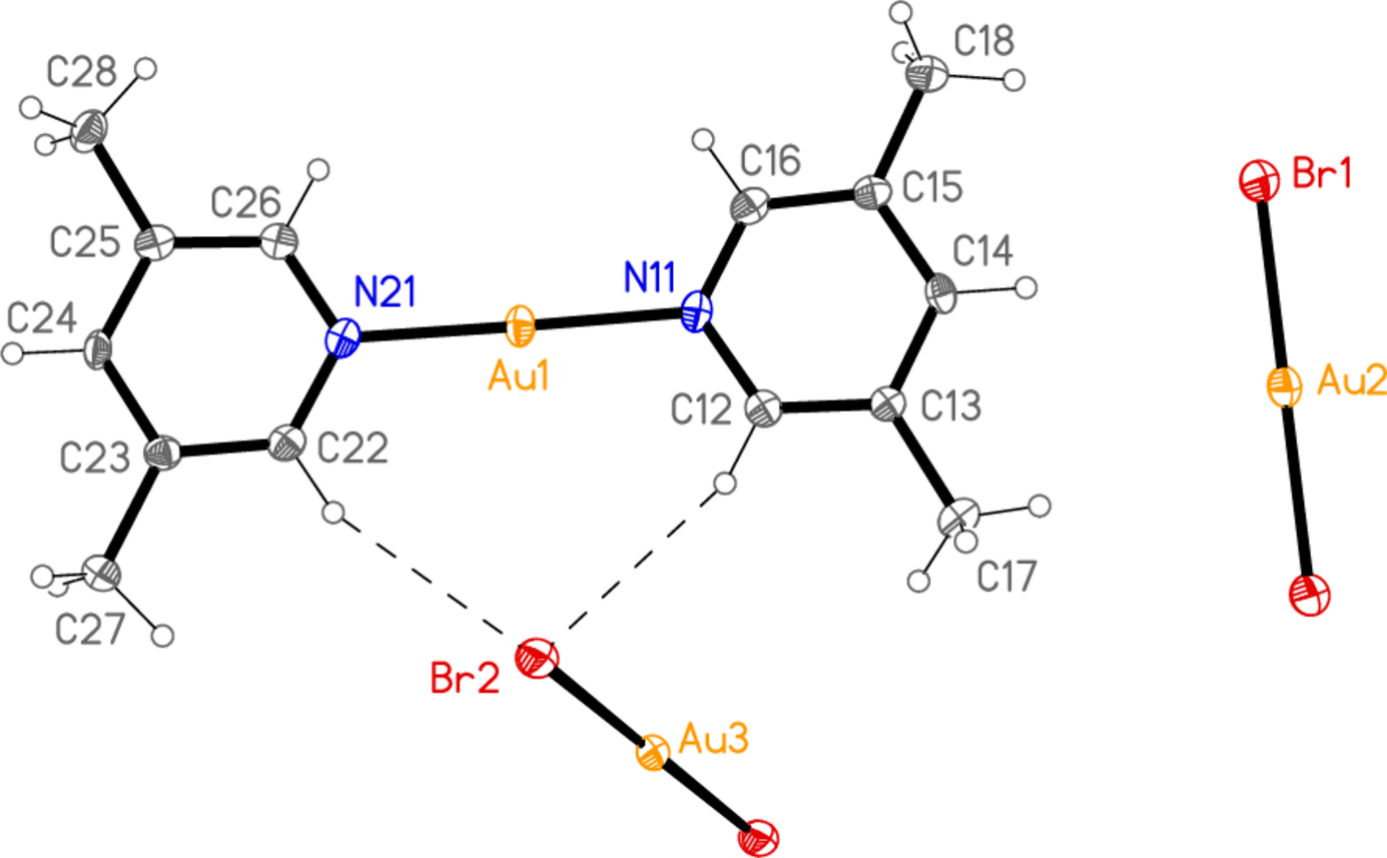
The structure of compound **4** in the crystal, showing the asymmetric unit (labelled) extended by symmetry. Ellipsoids correspond to 50% probability levels. Dashed lines indicate short H⋯Br contacts.

The structure of bis­(3,5-di­methyl­pyridine)­gold(I) di­chlorido­aurate(I) (**3**), which crystallizes in space group *P*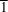 with *Z* = 2, is shown in Fig. 3 ▸, with selected dimensions in Table 3 ▸. The cation lies on a general position, and there are two independent anions in which the gold atoms lie on inversion centres. There are no aurophilic contacts within the asymmetric unit. The inter­planar angle between the rings is 8.61 (9)°, which corresponds to an out-of-plane bend about the gold atom rather than a mutual rotation around the N—Au—N coordination axis. Bis(3,5-di­methyl­pyridine)­gold(I) di­bromido­aurate(I) (**4**; Fig. 4 ▸, Table 4 ▸) is isotypic to **3**; its inter­planar angle is 7.8 (1)°.

**Figure 5:**
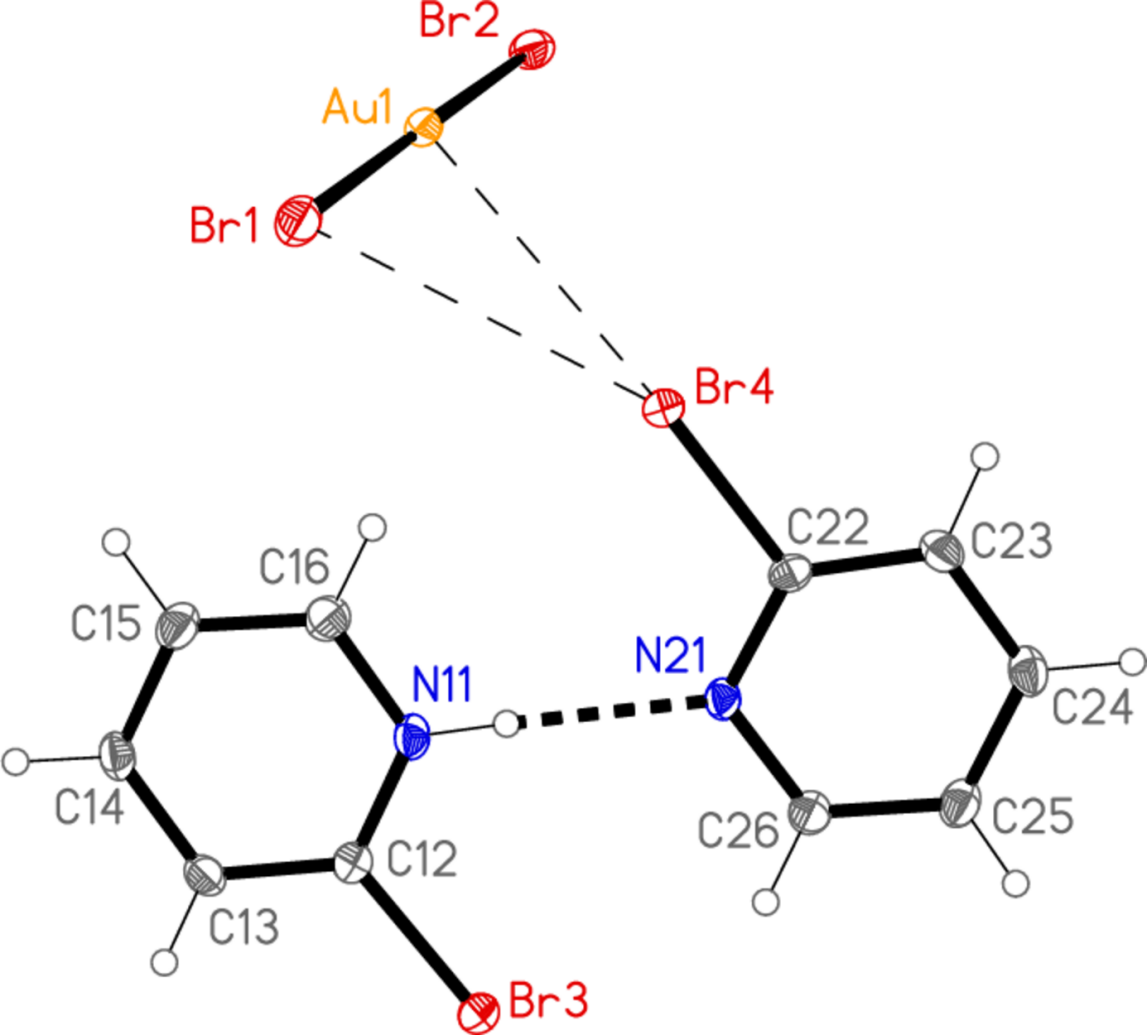
The structure of compound **5** in the crystal. Ellipsoids correspond to 50% probability levels. The dashed lines indicate a hydrogen bond (thick) and short Au⋯Br and Br⋯Br contacts (thin).

We did not succeed in making any further compounds (*cf*. Freytag & Jones, 2000 ▸) in which a bromo­pyridine was coordinated to gold. Attempts to make bis­(2-bromo­pyridine)­gold(I) di­bromido­aurate(I) (or the corresponding neutral mol­ecule) led instead to 2-bromo­pyridine 2-bromo­pyridinium di­bromido­aurate(I) (**5**; Fig. 5 ▸), possibly because of small amounts of adventitious water. Compound **5** crystallizes in space group *P*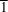 with *Z* = 2; all atoms lie on general positions. The 2-bromo­pyridinium cation is linked to the 2-bromo­pyridine mol­ecule by an N—H⋯N hydrogen bond. The NH hydrogen atom was refined freely, and there are no signs of disorder of this atom. The ring angle at the nitro­gen atom is 5° larger for the cation than for the neutral mol­ecule (Table 5 ▸), and the inter­planar angle between the rings is 1.9 (2)°.

The bond lengths and angles in compounds **1**–**5** may be considered normal. The [*L*_2_Au]^+^ cations and the [Au*X*_2_]^−^ anions are linear at the gold atom, with maximum deviations of *ca* 3.5° for the cations of **3** and **4**. The six independent Au—Br bond lengths range from 2.3775 (5) to 2.3951 (4) Å. The Au—N bond lengths in **1**–**4** are almost constant at 2.012 (3)–2.027 (3) Å, as are the C—N—C angles at 118.9 (3)–119.7 (3)°, appreciably wider than in free pyridine (116.4–116.8° in four independent mol­ecules; Mootz & Wussow, 1981 ▸).

The related structure of 3-bromo­pyridine 3-bromo­pyridinium di­bromido­aurate(I) (**6**) was determined; it crystallizes in space group *C*2/*c* with *Z* = 4, with the gold atom on an inversion centre at (0.25, 0.25, 0.5). However, the NH hydrogen atom is disordered over a twofold axis connecting both bromo­pyridine residues (and was refined freely as a ‘half’ hydrogen atom). The *U* values of the bromo­pyridine site were somewhat high, which probably indicates that this residue is also disordered, over two closely adjacent positions corresponding to a superposition of the cation and the neutral mol­ecule. For this reason, we do not discuss this structure here, but have deposited it (with all faults) for the inter­ested reader (Döring & Jones, 2024*c* ▸).

## 3. Supra­molecular features

Hydrogen bonds, mostly of the type C—H⋯*X*, for all structures are given in Tables 6 ▸–10 ▸ ▸ ▸ ▸. These include several borderline cases that are not discussed explicitly.

**Figure 6:**
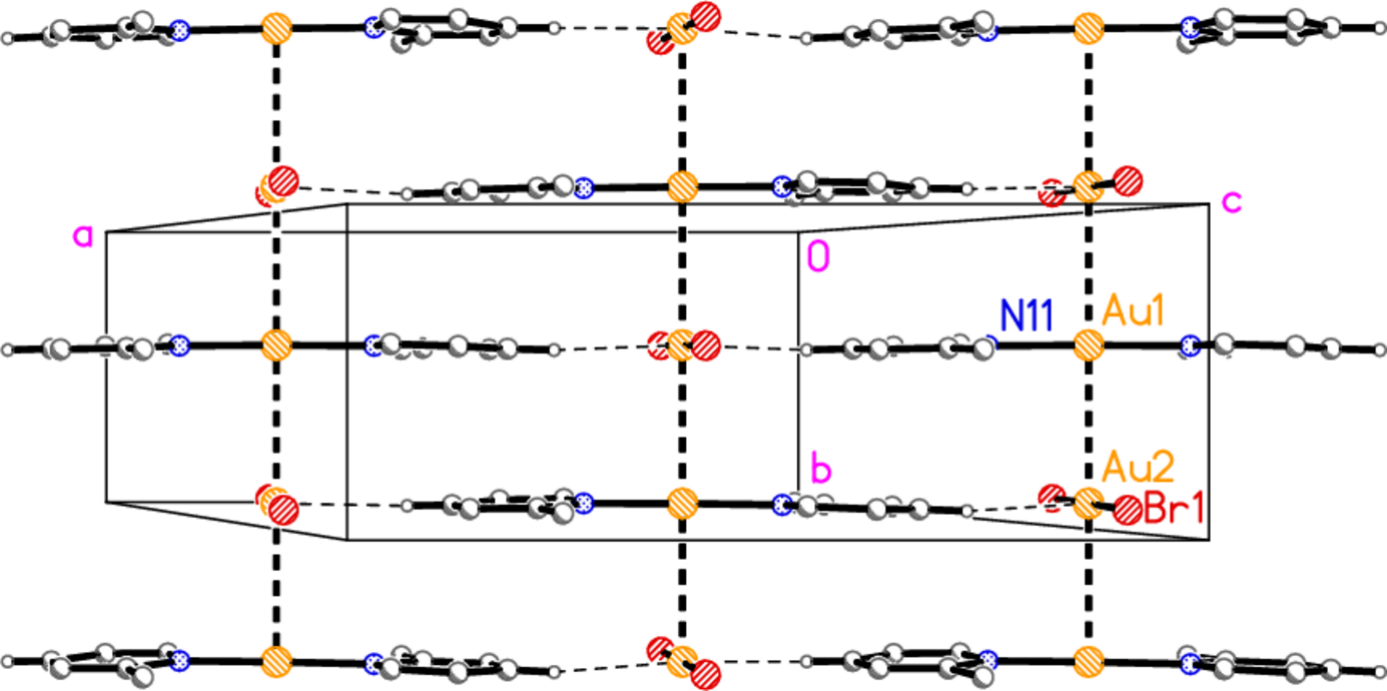
Packing diagram of compound **1** viewed perpendicular to the *ab* plane in the region *z* ≃ 0.75. Dashed lines indicate Au⋯Au contacts (thick) or H⋯Au contacts (thin). Atom labels indicate the asymmetric unit. In all packing diagrams, the hydrogen atoms not involved in significant contacts are omitted.

**Figure 7:**
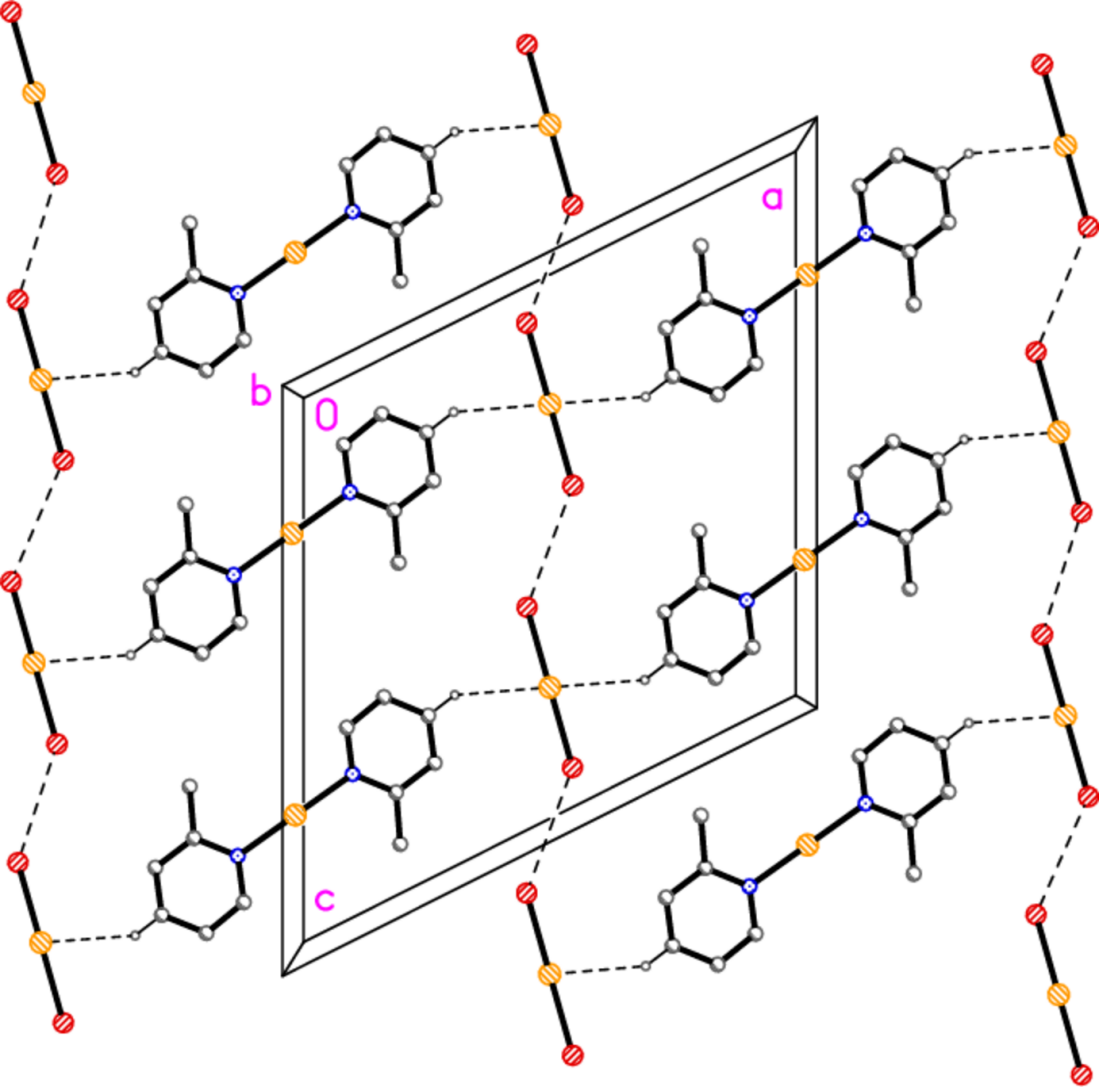
Packing diagram of compound **1** viewed perpendicular to the *bc* plane in the region *y* ≃ 0.5. Dashed lines indicate Br⋯Br or H⋯Au contacts.

Compound **1**: A second aurophilic contact, Au1⋯Au2(*x*, −1 + *y*, *z*) = 3.1937 (4) Å, connects the gold atoms to form infinite chains of alternating anions and cations parallel to the *b* axis (Fig. 6 ▸). The Au⋯Au⋯Au angles are exactly 180° by symmetry. Adjacent chains are linked by the short contact H14⋯Au2, 2.75 Å, which could be classed as a hydrogen bond with gold as acceptor, to complete a layer structure parallel to the *ab* plane; for a detailed discussion of H⋯Au hydrogen bonds, see Schmidbaur (2019 ▸) and Schmidbaur *et al.* (2014 ▸). An alternative layer structure, parallel to the *ac* plane, is shown in Fig. 7 ▸; it involves the H⋯Au contacts and also borderline Br1⋯Br1′ contacts of 3.8543 (9) Å over inversion centres. The *C*-centring operator can be seen to move (*e.g.*) Au2 by **b**/2 into the paper and **a**/2 diagonally in the plane of the paper, thus placing it under Au1 to propagate the Au⋯Au chain.

**Figure 8:**
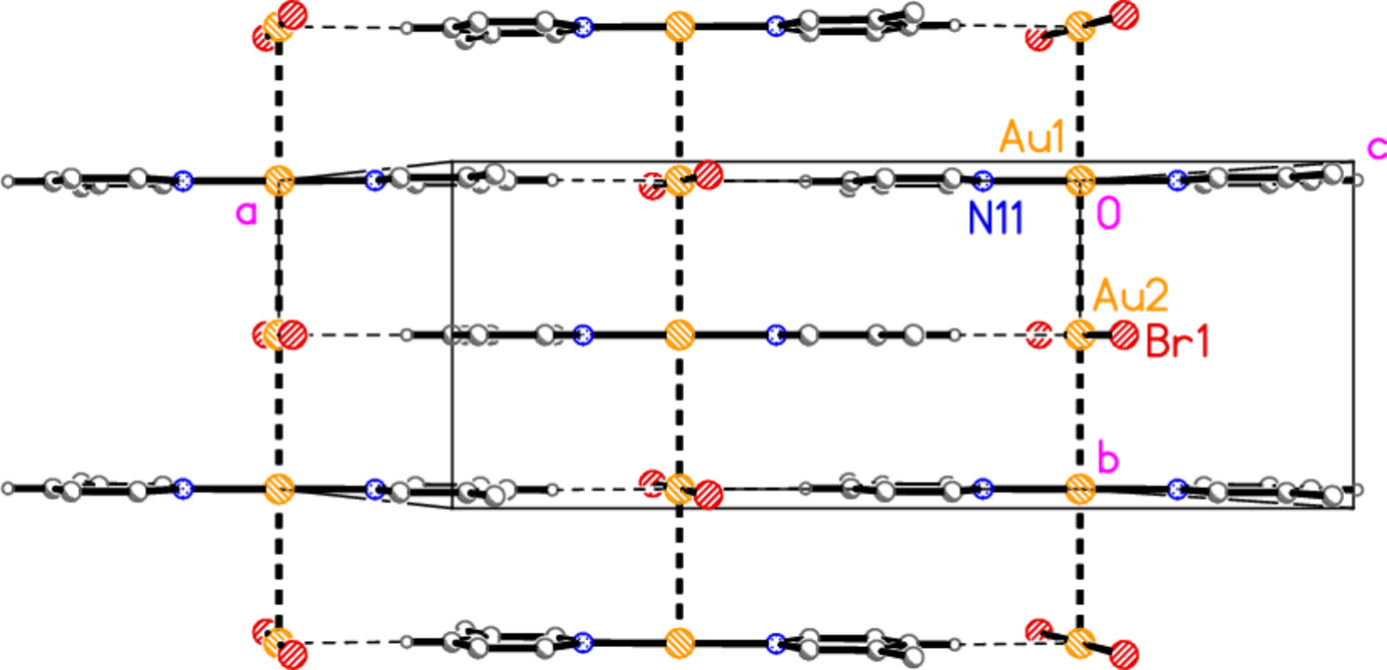
Packing diagram of compound **2** viewed perpendicular to the *ab* plane in the region *z* ≃ 0. Dashed lines indicate Au⋯Au contacts (thick) or H⋯Au contacts (thin). Atom labels indicate the asymmetric unit. Hydrogen atoms not involved in H⋯Au contacts are omitted.

**Figure 9:**
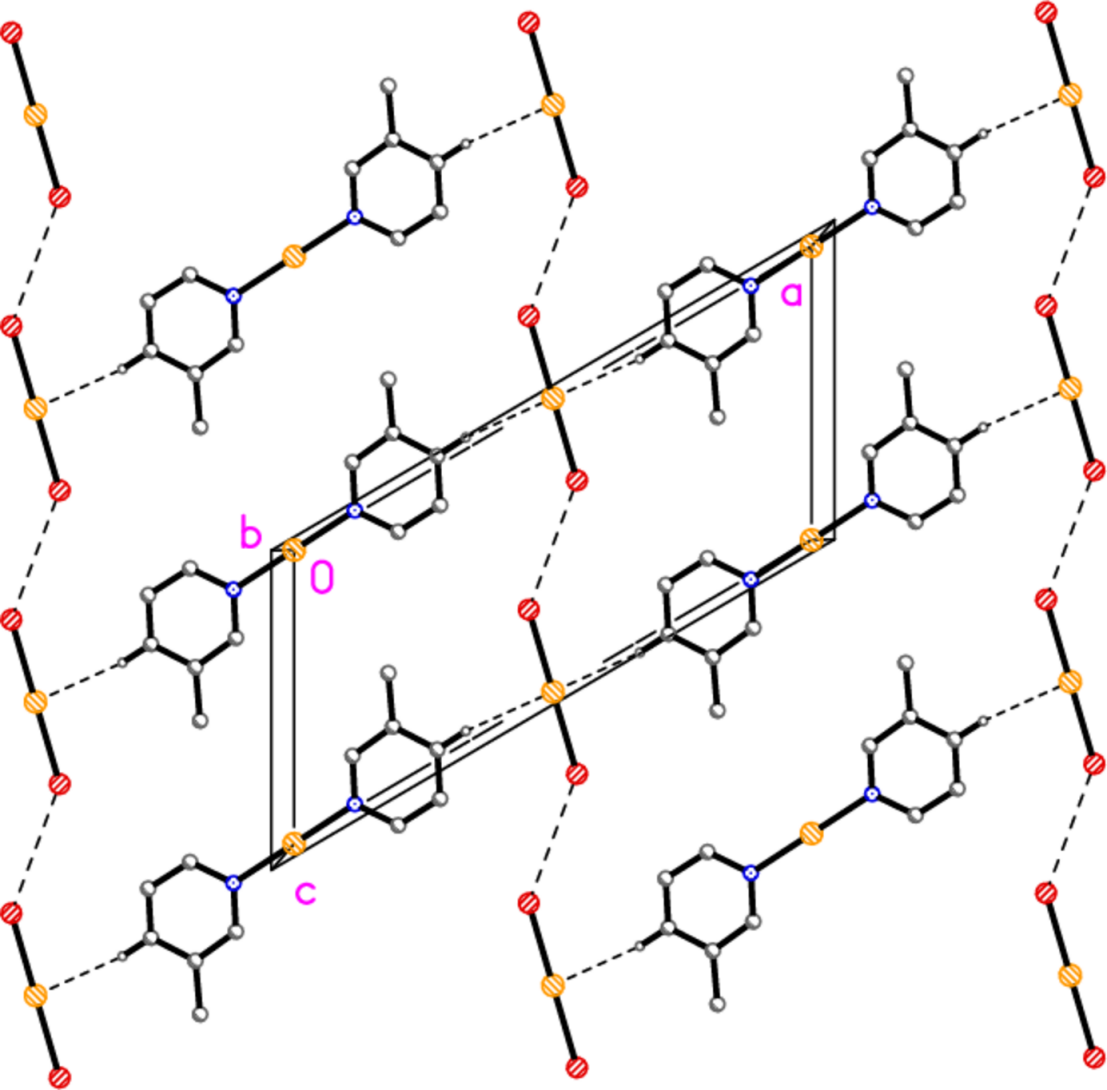
Packing diagram of compound **2** viewed perpendicular to the *bc* plane in the region *y* ≃ 0. Dashed lines indicate Br⋯Br or H⋯Au contacts.

Compound **2**: The packing is closely related to that of **1**. The aurophilic contacts are now equivalent and again connect the gold atoms to form infinite chains parallel to the *b* axis (Fig. 8 ▸). Adjacent chains are again linked by the short contact H14⋯Au2 (2.66 Å) to complete the layer structure parallel to the *ab* plane. The packing in layers parallel to the *ac* plane is also repeated, but the *c* axis is halved, so that adjacent cations (vertically displaced in Fig. 9 ▸) are translationally equivalent. The Br1⋯Br1′ contact is 3.8489 (7) Å *via* the operator −*x*, 1 − *y*, 1 − *z*.

The close similarity between Figs. 7 ▸ and 9 ▸ is evident. The structures of compounds **1** and **2** are effectively the same (except for the position of the methyl substituent and the small shifts associated with this), except that **1** has the higher formal symmetry. The usage of the term ‘isostructural’ in the crystallographic literature has often been inconsistent, but previously one might have defined the two structures as (nearly) isostructural (closely similar connectivity including the secondary contacts) but not isotypic (because of the different cells and space group). The IUCr (2019 ▸) has however defined the terms ‘isostructural’ and ‘isotypic’ as synonymous: ‘Two crystals are said to be *isostructural* if they have the same structure, but not necessarily the same cell dimensions nor the same chemical composition, and with a ‘comparable’ variability in the atomic coordinates to that of the cell dimensions and chemical composition … One also speaks of *isostructural series*, or of *isostructural polymorphs* or *isostructural phase transitions*. The term isotypic is synonymous with isostructural’ (their italics). Bombicz (2024 ▸) has recently commented: ‘… the definition of isostructurality is not explicit about several issues. Are the corresponding structures required to have the same stoichiometry, *Z*′, symmetry elements and the same space group?’, and we have pointed out the presence of some significant differences in formally isotypic structures (Upmann *et al.*, 2024 ▸). We too would suggest that the definitions need further amendment and/or clarification.

**Figure 10:**
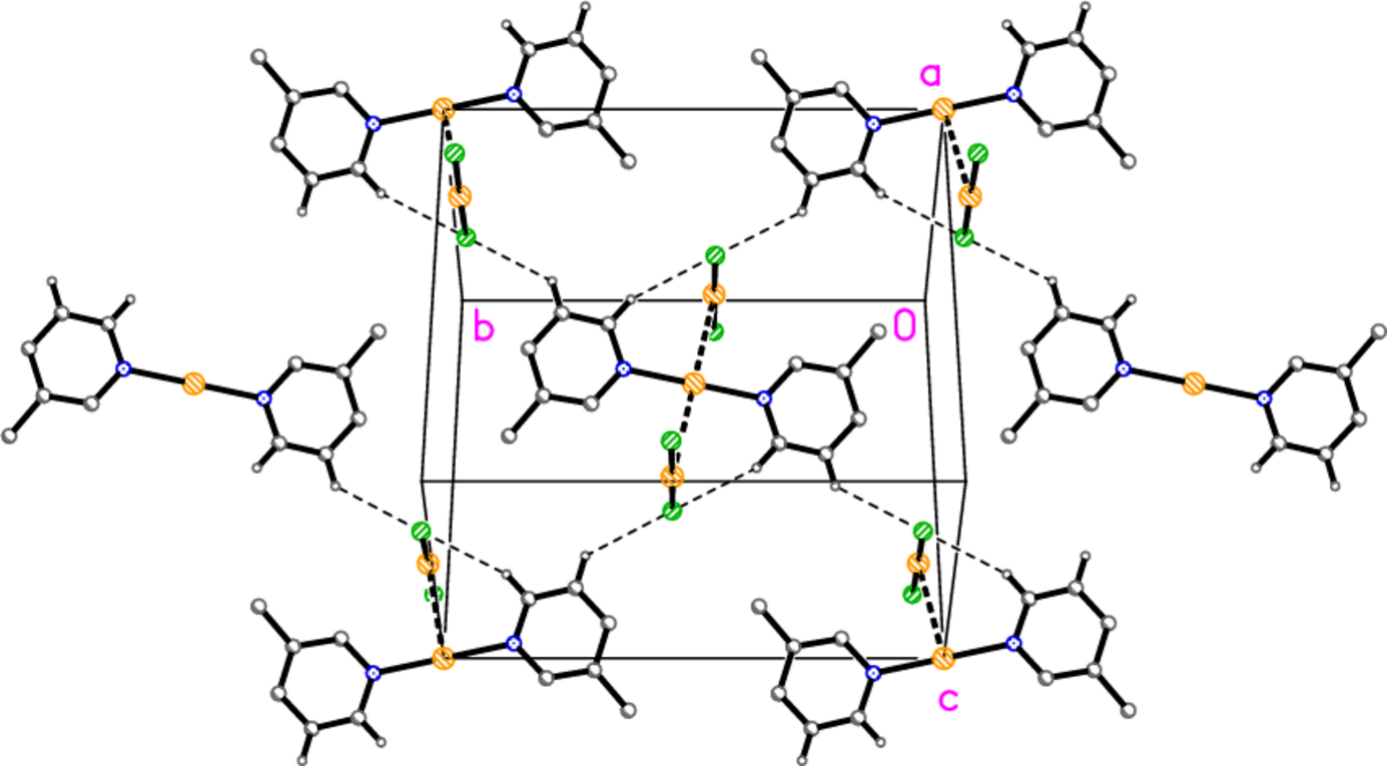
Packing diagram of bis­(3-methyl­pyridine)­gold(I) di­chlorido­aurate(I) (Jones & Ahrens, 1998 ▸) viewed perpendicular to (101). Dashed lines indicate H⋯Cl (thin) or Au⋯Au (thick) contacts. The Au⋯Au⋯Au chains propagate parallel to the *c* axis, and only short sections of these chains are visible in this view.

The packing of bis­(3-methyl­pyridine)­gold(I) di­chlorido­aurate(I) (Jones & Ahrens, 1998 ▸) is not closely related to those of compounds **1** and **2**, although it too crystallizes in a *C*-centred monoclinic space group (*C*2/*c*). The chains of alternating cations and anions parallel to the *c* axis were described in the original publication. However, at the time ‘weak’ hydrogen bonds were not generally discussed, so we rectify that omission here. Fig. 10 ▸ shows the formation of a layer structure parallel to (101), whereby two ‘weak’ H⋯Cl hydrogen bonds (2.71, 2.81 Å) connect the ions. In contrast to **1** and **2**, there are no very short and linear C—H⋯Au contacts.

**Figure 11:**
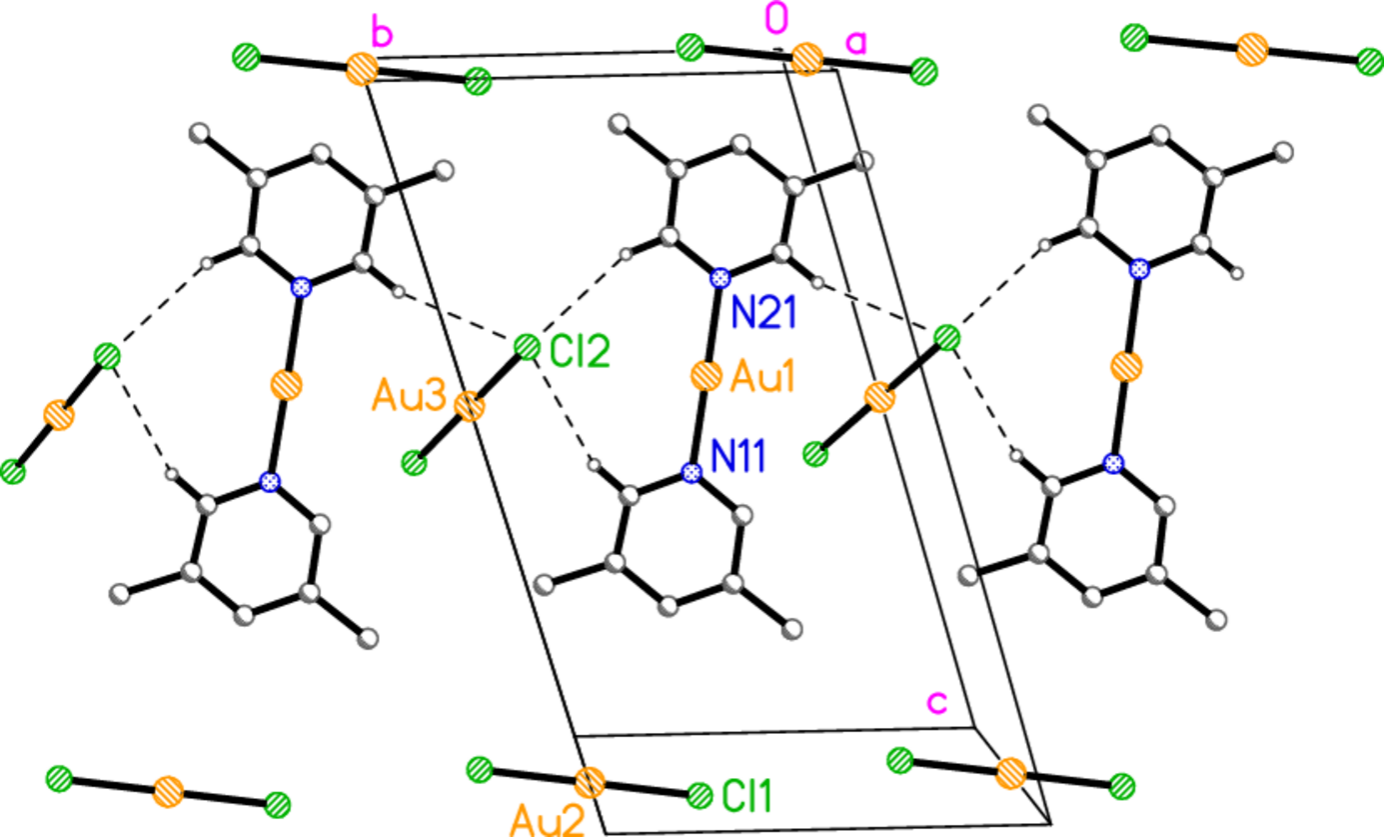
Packing diagram of compound **3** viewed perpendicular to (20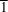), centred approximately on (1/4, 1/2, 1/2). Dashed lines indicate H⋯Cl contacts. Atom labels indicate the asymmetric unit. Hydrogen atoms not involved in H⋯Cl contacts are omitted.

**Figure 12:**
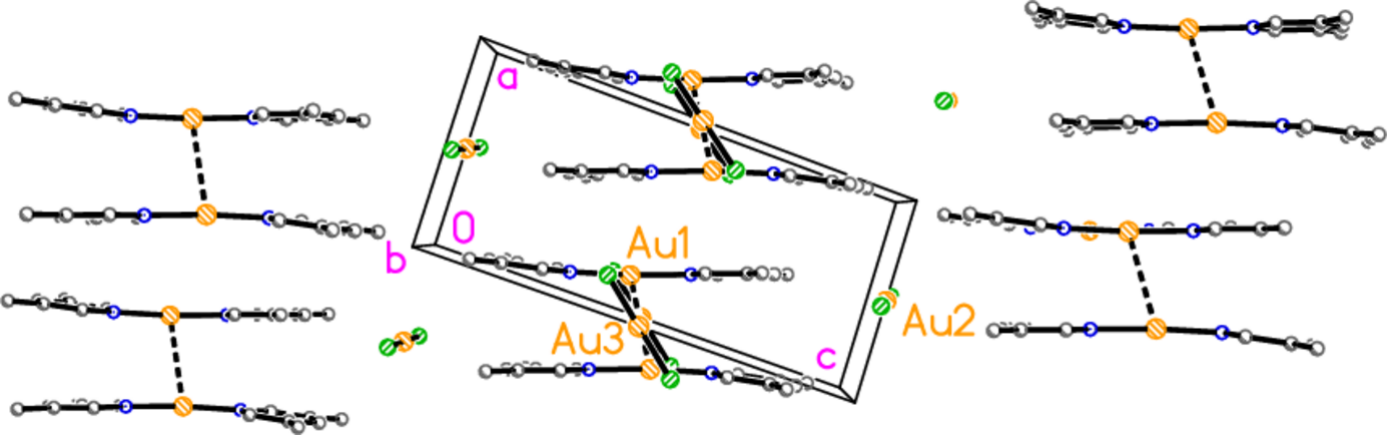
Packing diagram of compound **3** viewed parallel to the *b* axis. Hydrogen atoms are omitted. Dashed lines indicate Au⋯Au contacts; the Au3 anions left and right have been omitted to show these contacts more clearly.

Compound **3**: The shortest contacts between residues, H12⋯Cl2 and H22⋯Cl2 (Table 8 ▸) lie within the asymmetric unit and are shown in Fig. 3 ▸. An aurophilic contact Au1⋯Au1(−*x*, 1 − *y*, 1 − *z*) of 3.3495 (3) Å connects the cations in pairs. Fig. 11 ▸ shows the association of cations and Au3 anions, which are connected by the three shortest H⋯Cl contacts (all to Cl2), to form a ribbon structure parallel to the *b* axis and lying in a plane parallel to (20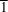). The shortest H⋯Cl1 contacts are > 2.94 Å and involve methyl hydrogens; they are not drawn explicitly. The view parallel to the *b* axis (Fig. 12 ▸) shows the aurophilic contacts between adjacent ribbons.

Compound **4** is isotypic to compound **3**, so that the packing diagrams are practically the same (but with Br instead of Cl). The Au1⋯Au1 contact is 3.4400 (3) Å.

**Figure 13:**
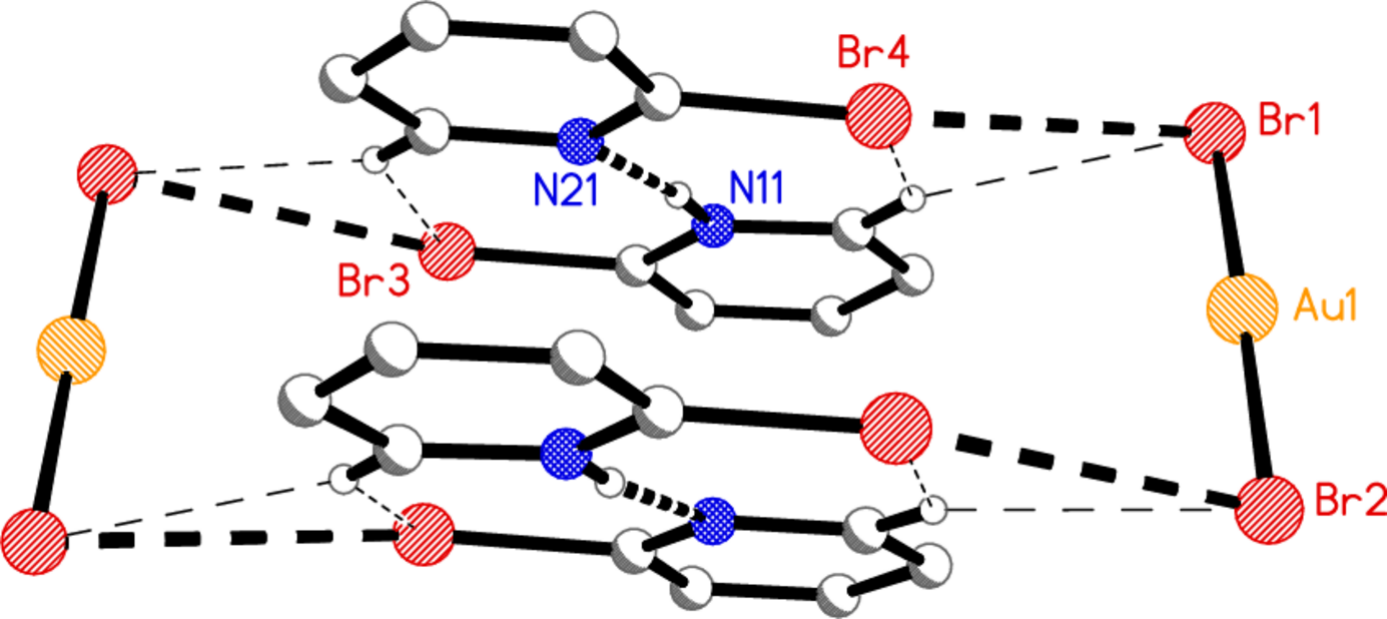
A dimeric unit of compound **5**. Hydrogen atoms not involved in H⋯Br contacts are omitted. Dashed lines indicate classical hydrogen bonds or Br⋯Br contacts (thick) or ‘weak’ H⋯Br hydrogen bonds (thin). The Au1⋯Br4 contacts (see Fig. 5 ▸) have also been omitted for clarity.

**Figure 14:**
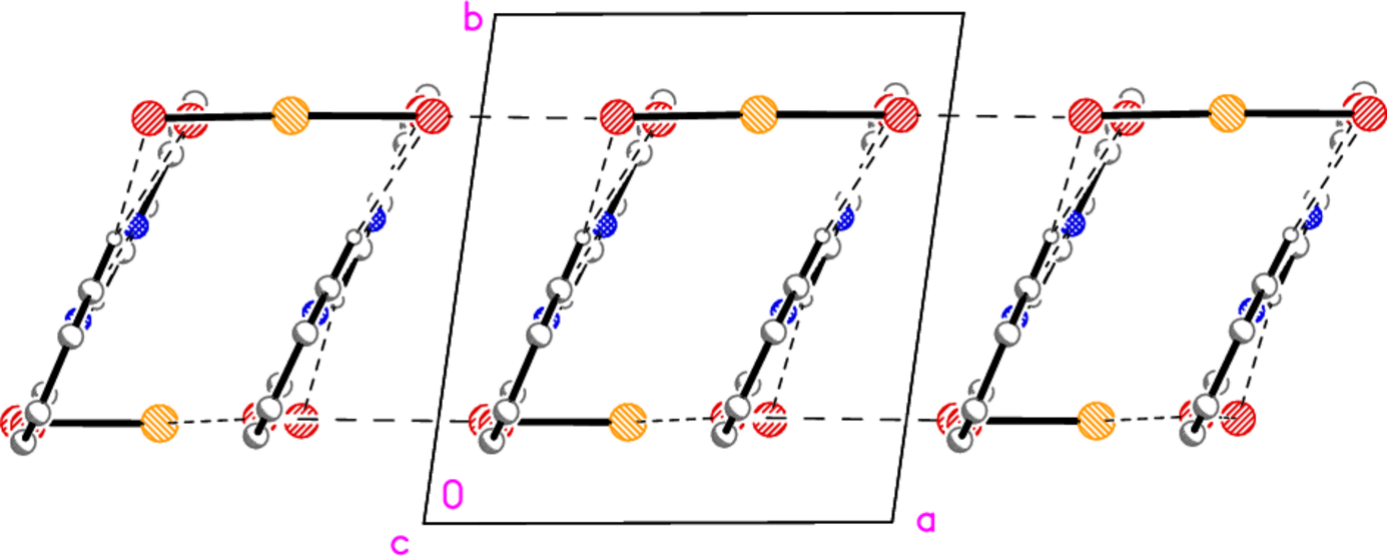
Several dimeric units of compound **5**, connected into chains parallel to the *a* axis by the Br2⋯Br4 contact. This view is a projection parallel to the *c* axis.

Compound **5**: Several short contacts lie within the asymmetric unit; Br1⋯Br4 = 3.6947 (5) Å and Au1⋯Br4 = 3.5636 (4) Å are shown explicitly in Fig. 5 ▸, where the probable ‘weak’ hydrogen bonds H16⋯Br4, H16⋯Br1 and H26⋯Br3 (Table 10 ▸) are not drawn but can be easily recognized. The inversion operator links two formula units (Fig. 13 ▸) involving the further short contact Br2⋯Br3 of 3.4720 (5) Å. The next shortest Br⋯Br contact is Br2⋯Br4(−1 + *x*, *y*, *z*) = 3.7614 (5) Å, which links the dimers parallel to the *a* axis (Fig. 14 ▸). The Br⋯Br contacts may be classed as ‘halogen bonds’ (see *e.g.* Metrangolo *et al.*, 2008 ▸).

## 4. Database survey

The searches employed the routine ConQuest (Bruno *et al.*, 2002 ▸), part of Version 2024.1.0 of the Cambridge Structural Database (Groom *et al.*, 2016 ▸).

A search for all gold(I) complexes involving pyridines (any substitution, but no fused rings) led to 116 hits. The average angle at nitro­gen was 118.4 (17) ° for 187 values and the average Au—N bond length was 2.058 (30) Å, but the latter values showed a considerable spread (2.003–2.137 Å); as would be expected from the known *trans* influences, the shortest Au—N bonds were observed *trans* to halogen or nitro­gen donors and the longest *trans* to phospho­rus donors. Two further ‘simple’ derivatives involving only alkyl­pyridine and halogenido ligands were found: bis­(2,6-di­methyl­pyridine)­gold(I) di­chlorido­aurate(I), which displays the known structure type with alternating cations and anions connected by Au⋯Au contacts (3.334 and 3.328 Å; refcode BUVTUI, Hashmi *et al.*, 2010 ▸) and chlorido­(4-ethyl­pyridine)­gold(I), a mol­ecular structure without aurophilic contacts (ESITAE; Hobbollahi *et al.*, 2019 ▸).

## 5. Synthesis and crystallization

*Bis(2-methyl­pyridine)­gold(I) di­bromido­daurate(I)* (**1**): 55 mg (0.104 mmol) of (tht)AuBr_3_ (tht = tetra­hydro­thio­phene) were dissolved in 2 mL of 2-methyl­pyridine. The clear, deep red solution was divided amongst five ignition tubes, overlayered with the five precipitants *n*-pentane, *n*-heptane, diethyl ether, diisopropyl ether and petroleum ether (b.p. 313–333 K) and transferred to a refrigerator (276 K). A red oil formed, in which some colourless blocks of compound **1** were observed and removed for investigation. The measured crystal was taken from the tube with *n*-pentane as precipitant. Elemental analysis [%]: calculated C 19.48, H 1.91, N 3.79; found C 18.89, H 1.89, N 3.91. This synthesis was intended to lead to tri­bromido­(2-methyl­pyridine)­gold(III), which we later obtained in crystalline form using a different method (to be published), and which probably corresponds to the red oil. We can see no obvious reason for the observed reduction to gold(I); the 2-methyl­pyridine had been recently redistilled.

*Bis(3-methyl­pyridine)­gold(I) di­bromido­aurate(I)* (**2**): 45.6 mg (0.125 mmol) of (tht)AuBr were dissolved in 2 mL of 3-picoline. The solution was treated as above. Compound **2** was obtained as colourless blocks. The measured crystal was taken from the tube with diisopropyl ether as precipitant. Elemental analysis [%]: calculated C 19.48, H 1.91, N 3.79; found C 19.29, H 1.99, N 3.86.

*Bis(3,5-di­methyl­pyridine)­gold(I) di­chlorido­aurate(I)* (**3**): 40 mg (0.125 mmol) of (tht)AuCl were dissolved in 2 mL of 3,5-di­methyl­pyridine by sonication. The solution was treated as above. Compound **3** was obtained as colourless plates. The measured crystal was taken from the tube with *n*-heptane as precipitant. Elemental analysis [%]: calculated C 24.76, H 2.67, N 4.13; found C 25.09, H 2.80, N 4.06.

*Bis(3,5-di­methyl­pyridine)­gold(I) di­bromido­aurate(I)* (**4**): 45.6 mg (0.125 mmol) of (tht)AuBr were sonicated with 2 mL of 3,5-di­methyl­pyridine. The solution was filtered and then treated as above. Compound **4** was obtained as colourless blocks. The measured crystal was taken from the tube with *n*-pentane as precipitant. Elemental analysis [%]: calculated C 21.89, H 2.36, N 3.65; found C 21.73, H 2.38, N 3.70.

*2-Bromo­pyridine 2-bromo­pyridinium di­bromido­aurate(I)* (**5**): 45.6 mg (0.125 mmol) of (tht)AuBr were dissolved in 2 mL of 2-bromo­pyridine. The solution was treated as above. Compound **5** was obtained as colourless blocks. The measured crystal was taken from the tube with diethyl ether as precipitant. Elemental analysis [%]: calculated C 17.82, H 1.35, N 4.16; found C 17.79, H 1.35, N 4.04.

*3-Bromo­pyridine 3-bromo­pyridinium di­bromido­aurate(I)* (**6**): 45.6 mg (0.125 mmol) of (tht)AuBr were dissolved in 2 mL of 3-bromo­pyridine. The solution was treated as above. Compound **6** was obtained as colourless needles. The measured crystal was taken from the tube with petroleum ether as precipitant. Elemental analysis [%]: calculated C 17.83, H 1.35, N 4.16; found C 16.74, H 1.18, N 3.90.

## 6. Refinement

Details of the measurements and refinements are given in Table 11 ▸. Structures were refined anisotropically on *F*^2^. For compound **5**, the NH hydrogen atom was refined freely. Aromatic hydrogens were included at calculated positions and refined using a riding model with C—H = 0.95 Å. Methyl groups were included as idealised rigid groups with C—H = 0.98 Å and H—C—H = 109.5°, and were allowed to rotate but not tip (command AFIX 137). *U* values of the hydrogen atoms were fixed at 1.5 × *U*_eq_ of the parent carbon atoms for methyl groups and 1.2 × *U*_eq_ of the parent carbon atoms for other hydrogens. For compounds **1**, **2** and **3**, three, one and one badly fitting reflection(s), respectively, were omitted.

Special aspects for compound **2**: The structure was refined in a non-reduced setting of *C*2/*m* to facilitate comparison with structure **1** (see *Supra­molecular features*), The reorientation matrix −1 0 −2 / 0 −1 0 / 0 0 1 converts the cell to a *C*-centred cell with *a* = 16.460, *b* = 6.441, *c* = 8.192 Å and a lower β angle of 118.72°, whereas the matrix 0 0 −1 / 0 −1 0 / −1 0 −1 leads to an *I*-centred cell with *a* = 8.192, *b* = 6.441, *c* = 14.437 Å and β = 91.12°. The carbon atom of the methyl group (C17) lies in a mirror plane; its hydrogen atoms (one in the mirror plane and one on a general position) were refined freely, but with C—H distances restrained to be approximately equal (command SADI).

## Supplementary Material

Crystal structure: contains datablock(s) 1, 2, 3, 4, 5, global. DOI: 10.1107/S2056989024005437/yz2055sup1.cif

Structure factors: contains datablock(s) 1. DOI: 10.1107/S2056989024005437/yz20551sup2.hkl

Structure factors: contains datablock(s) 2. DOI: 10.1107/S2056989024005437/yz20552sup3.hkl

Structure factors: contains datablock(s) 3. DOI: 10.1107/S2056989024005437/yz20553sup4.hkl

Structure factors: contains datablock(s) 4. DOI: 10.1107/S2056989024005437/yz20554sup5.hkl

Structure factors: contains datablock(s) 5. DOI: 10.1107/S2056989024005437/yz20555sup6.hkl

CCDC references: 2145201, 2145220, 2145219, 2145211, 2145205

Additional supporting information:  crystallographic information; 3D view; checkCIF report

## Figures and Tables

**Table 1:** Selected geometric parameters (Å, °) for **1**[Chem scheme1]

Au1—N11	2.027 (3)	Au1—Au2^i^	3.1937 (4)
Au1—Au2	3.1907 (4)	Au2—Br1	2.3951 (4)
			
N11—Au1—N11^ii^	179.35 (19)	Br1—Au2—Au1	90.282 (12)
N11—Au1—Au2	89.67 (9)	Br1—Au2—Au1^iii^	89.718 (12)
N11—Au1—Au2^i^	90.33 (9)	Au1—Au2—Au1^iii^	180.0
Au2—Au1—Au2^i^	180.0	C16—N11—C12	119.7 (3)
Br1—Au2—Br1^ii^	179.44 (2)		
			
N11^ii^—Au1—Au2—Br1	70.36 (9)	N11—Au1—Au2—Br1	−109.64 (9)

Symmetry codes: (i) 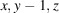; (ii) 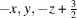; (iii) 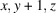.

**Table 2:** Selected geometric parameters (Å, °) for **2**[Chem scheme1]

Au1—N11	2.021 (3)	Au2—Br1	2.3906 (3)
Au1—Au2	3.2205 (1)		
			
N11—Au1—N11^i^	180.0	Br1—Au2—Au1	90.0
Au2—Au1—Au2^ii^	180.0	Au1—Au2—Au1^iv^	180.0
Br1^iii^—Au2—Br1	180.0	C16—N11—C12	118.9 (3)
			
N11—Au1—Au2—Br1	−106.73 (8)	N11^i^—Au1—Au2—Br1	73.27 (8)

Symmetry codes: (i) 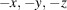; (ii) 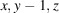; (iii) 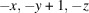; (iv) 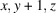.

**Table 3:** Selected geometric parameters (Å, °) for **3**[Chem scheme1]

Au1—N11	2.013 (3)	Au2—Cl1	2.2551 (9)
Au1—N21	2.016 (3)	Au3—Cl2	2.2617 (9)
Au1—Au1^i^	3.3495 (3)		
			
N11—Au1—N21	176.33 (10)	Cl2—Au3—Cl2^iii^	180.0
N11—Au1—Au1^i^	75.62 (7)	C16—N11—C12	119.4 (3)
Cl1—Au2—Cl1^ii^	180.0	C26—N21—C22	119.0 (3)

Symmetry codes: (i) 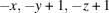; (ii) 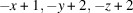; (iii) 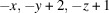.

**Table 4:** Selected geometric parameters (Å, °) for **4**[Chem scheme1]

Au1—N11	2.012 (3)	Au2—Br1	2.3775 (5)
Au1—N21	2.016 (4)	Au3—Br2	2.3789 (5)
Au1—Au1^i^	3.4400 (3)		
			
N11—Au1—N21	176.50 (13)	C12—N11—C16	119.1 (4)
Br1—Au2—Br1^ii^	180.0	C26—N21—C22	119.1 (4)
Br2^iii^—Au3—Br2	180.0		

Symmetry codes: (i) 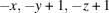; (ii) 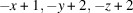; (iii) 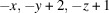.

**Table 5:** Selected geometric parameters (Å, °) for **5**[Chem scheme1]

Au1—Br1	2.3790 (4)	N11—C16	1.346 (4)
Au1—Br2	2.3851 (4)	N21—C22	1.327 (4)
N11—C12	1.345 (4)	N21—C26	1.352 (4)
			
Br1—Au1—Br2	178.713 (12)	C22—N21—C26	116.2 (3)
C12—N11—C16	121.2 (3)		

**Table 6:** Hydrogen-bond geometry (Å, °) for **1**[Chem scheme1]

*D*—H⋯*A*	*D*—H	H⋯*A*	*D*⋯*A*	*D*—H⋯*A*
C17—H17*B*⋯Br1^iv^	0.98	3.01	3.971 (4)	167
C14—H14⋯Au2^v^	0.95	2.75	3.581 (4)	147

Symmetry codes: (iv) 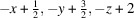; (v) 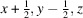.

**Table 7:** Hydrogen-bond geometry (Å, °) for **2**[Chem scheme1]

*D*—H⋯*A*	*D*—H	H⋯*A*	*D*⋯*A*	*D*—H⋯*A*
C15—H15⋯Br1^v^	0.95	2.97	3.822 (3)	151
C14—H14⋯Au2^vi^	0.95	2.66	3.604 (3)	171

Symmetry codes: (v) 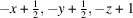; (vi) 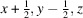.

**Table 8:** Hydrogen-bond geometry (Å, °) for **3**[Chem scheme1]

*D*—H⋯*A*	*D*—H	H⋯*A*	*D*⋯*A*	*D*—H⋯*A*
C18—H18*C*⋯Cl1	0.98	2.95	3.881 (4)	160
C28—H28*B*⋯Cl1^iv^	0.98	2.96	3.934 (4)	173
C12—H12⋯Cl2	0.95	2.78	3.689 (3)	160
C22—H22⋯Cl2	0.95	2.77	3.670 (3)	159
C26—H26⋯Cl2^v^	0.95	2.86	3.787 (3)	164
C26—H26⋯Au3^v^	0.95	3.01	3.844 (3)	148
C26—H26⋯Au3^i^	0.95	3.01	3.844 (3)	148
C17—H17*A*⋯Cl2^vi^	0.98	3.00	3.941 (4)	162

Symmetry codes: (i) 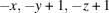; (iv) 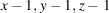; (v) 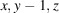; (vi) 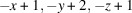.

**Table 9:** Hydrogen-bond geometry (Å, °) for **4**[Chem scheme1]

*D*—H⋯*A*	*D*—H	H⋯*A*	*D*⋯*A*	*D*—H⋯*A*
C14—H14⋯Br1	0.95	3.08	3.961 (4)	155
C18—H18*B*⋯Br1	0.98	3.08	4.008 (5)	159
C27—H27*B*⋯Br1^i^	0.98	3.06	4.029 (5)	172
C27—H27*C*⋯Br1^iv^	0.98	3.10	3.955 (5)	147
C28—H28*A*⋯Br1^v^	0.98	3.05	4.026 (5)	173
C12—H12⋯Br2	0.95	2.86	3.771 (4)	160
C17—H17*A*⋯Br2^vi^	0.98	3.02	3.911 (4)	151
C22—H22⋯Br2	0.95	2.86	3.755 (4)	158
C26—H26⋯Br2^vii^	0.95	3.01	3.933 (4)	166

Symmetry codes: (i) 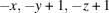; (iv) 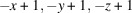; (v) 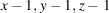; (vi) 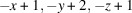; (vii) 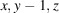.

**Table 10:** Hydrogen-bond geometry (Å, °) for **5**[Chem scheme1]

*D*—H⋯*A*	*D*—H	H⋯*A*	*D*⋯*A*	*D*—H⋯*A*
N11—H11⋯N21	0.87 (4)	1.99 (4)	2.861 (4)	174 (4)
C16—H16⋯Br1	0.95	2.88	3.675 (3)	142
C13—H13⋯Br2^i^	0.95	2.93	3.853 (3)	163
C26—H26⋯Br2^ii^	0.95	2.99	3.843 (3)	151
C26—H26⋯Br3	0.95	2.87	3.616 (3)	136
C16—H16⋯Br4	0.95	2.83	3.584 (3)	137

Symmetry codes: (i) 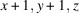; (ii) 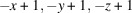.

**Table 11:** Experimental details

	**1**	**2**	**3**	**4**	**5**
Crystal data					
Chemical formula	[Au(C_6_H_7_N)_2_][AuBr_2_]	[Au(C_6_H_7_N)_2_][AuBr_2_]	[Au(C_7_H_9_N)_2_][AuCl_2_]	[Au(C_7_H_9_N)_2_][AuBr_2_]	(C_5_H_5_BrN)[AuBr_2_]·C_5_H_4_BrN
*M*_r_	740.00	740.00	679.14	768.06	673.80
Crystal system, space group	Monoclinic, *C*2/*c*	Monoclinic, *C*2/*m*	Triclinic, *P* 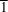	Triclinic, *P* 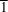	Triclinic, *P* 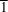
Temperature (K)	100	100	100	100	100
*a*, *b*, *c* (Å)	16.3717 (7), 6.3844 (3), 16.1850 (8)	16.7380 (6), 6.44097 (13), 8.1923 (3)	6.7718 (3), 8.5627 (5), 15.1064 (8)	6.8343 (2), 8.6676 (3), 15.4049 (6)	7.9931 (4), 8.4672 (3), 11.3923 (5)
α, β, γ (°)	90, 116.649 (6), 90	90, 120.415 (5), 90	105.356 (5), 90.788 (4), 96.311 (4)	105.720 (3), 90.741 (3), 98.242 (3)	87.202 (4), 74.635 (4), 81.406 (4)
*V* (Å^3^)	1512.00 (13)	761.65 (5)	838.71 (8)	868.05 (6)	735.09 (6)
*Z*	4	2	2	2	2
Radiation type	Mo *K*α	Mo *K*α	Mo *K*α	Mo *K*α	Mo *K*α
μ (mm^−1^)	24.65	24.47	17.78	21.48	20.86
Crystal size (mm)	0.25 × 0.06 × 0.02	0.15 × 0.15 × 0.10	0.15 × 0.15 × 0.01	0.15 × 0.10 × 0.03	0.15 × 0.10 × 0.04

Data collection					
Diffractometer	Oxford Diffraction Xcalibur, Eos	Oxford Diffraction Xcalibur, Eos	Oxford Diffraction Xcalibur, Eos	Oxford Diffraction Xcalibur, Eos	Oxford Diffrection Xcalibur, Eos
Absorption correction	Multi-scan (*CrysAlis PRO*; Rigaku OD, 2014 ▸)	Multi-scan (*CrysAlis PRO*; Rigaku OD, 2014 ▸)	Multi-scan (*CrysAlis PRO*; Rigaku OD, 2014 ▸)	Multi-scan (*CrysAlis PRO*; Rigaku OD, 2014 ▸)	Multi-scan (*CrysAlis PRO*; Rigaku OD, 2014 ▸)
*T*_min_, *T*_max_	0.275, 1.000	0.487, 1.000	0.330, 1.000	0.256, 1.000	0.241, 1.000
No. of measured, independent and observed [*I* > 2σ(*I*)] reflections	19772, 2268, 1793	25258, 1255, 1162	44852, 5018, 4253	47547, 5187, 4424	42320, 4361, 3917
*R*_int_	0.041	0.034	0.054	0.055	0.044
θ values (°)	θ_max_ = 30.9, θ_min_ = 2.8	θ_max_ = 30.9, θ_min_ = 2.8	θ_max_ = 31.0, θ_min_ = 2.5	θ_max_ = 30.9, θ_min_ = 2.5	θ_max_ = 30.9, θ_min_ = 2.4
(sin θ/λ)_max_ (Å^−1^)	0.722	0.723	0.724	0.722	0.723

Refinement					
*R*[*F*^2^ > 2σ(*F*^2^)], *wR*(*F*^2^), *S*	0.019, 0.041, 1.10	0.013, 0.029, 1.12	0.022, 0.040, 1.06	0.026, 0.054, 1.05	0.023, 0.048, 1.07
No. of reflections	2268	1255	5018	5187	4361
No. of parameters	84	65	189	188	158
No. of restraints	0	1	0	0	0
H-atom treatment	H-atom parameters constrained	H atoms treated by a mixture of independent and constrained refinement	H-atom parameters constrained	H-atom parameters constrained	H atoms treated by a mixture of independent and constrained refinement
Δρ_max_, Δρ_min_ (e Å^−3^)	1.31, −1.16	1.02, −0.93	1.04, −0.87	1.24, −1.80	1.15, −1.49
Extinction method	None	*SHELXL2019/3* (Sheldrick, 2015 ▸), *F*_c_^*^ = *kF*_c_[1 + 0.001*xF*_c_^2^λ^3^/sin(2θ)]^−1/4^	*SHELXL2019/3* (Sheldrick, 2015 ▸), *F*_c_^*^ = *kF*_c_[1 + 0.001*xF*_c_^2^λ^3^/sin(2θ)]^−1/4^	None	None
Extinction coefficient	–	0.00089 (6)	0.00055 (6)	–	–

Computer programs: *CrysAlis PRO* (Rigaku OD, 2014 ▸), *SHELXS97* (Sheldrick, 2008 ▸), *SHELXL2019/3* (Sheldrick, 2015 ▸) and *XP* (Bruker, 1998 ▸).

## supplementary crystallographic information

### Bis(2-methylpyridine)gold(I) dibromidoaurate(I) (1) Crystal data

**Table:**

[Au(C_6_H_7_N)_2_][AuBr_2_]	*F*(000) = 1312
*M**_r_* = 740.00	*D*_x_ = 3.251 Mg m^−^^3^
Monoclinic, *C*2/*c*	Mo *K*α radiation, λ = 0.71073 Å
*a* = 16.3717 (7) Å	Cell parameters from 5716 reflections
*b* = 6.3844 (3) Å	θ = 2.8–30.0°
*c* = 16.1850 (8) Å	µ = 24.65 mm^−^^1^
β = 116.649 (6)°	*T* = 100 K
*V* = 1512.00 (13) Å^3^	Block, colourless
*Z* = 4	0.25 × 0.06 × 0.02 mm

### Bis(2-methylpyridine)gold(I) dibromidoaurate(I) (1) Data collection

**Table:**

Oxford Diffraction Xcalibur, Eos diffractometer	2268 independent reflections
Radiation source: Enhance (Mo) X-ray Source	1793 reflections with *I* > 2σ(*I*)
Graphite monochromator	*R*_int_ = 0.041
Detector resolution: 16.1419 pixels mm^-1^	θ_max_ = 30.9°, θ_min_ = 2.8°
ω scan	*h* = −23→23
Absorption correction: multi-scan (CrysAlisPro; Rigaku OD, 2014)	*k* = −8→9
*T*_min_ = 0.275, *T*_max_ = 1.000	*l* = −22→23
19772 measured reflections	

### Bis(2-methylpyridine)gold(I) dibromidoaurate(I) (1) Refinement

**Table:**

Refinement on *F*^2^	Primary atom site location: structure-invariant direct methods
Least-squares matrix: full	Secondary atom site location: difference Fourier map
*R*[*F*^2^ > 2σ(*F*^2^)] = 0.019	Hydrogen site location: inferred from neighbouring sites
*wR*(*F*^2^) = 0.041	H-atom parameters constrained
*S* = 1.10	*w* = 1/[σ^2^(*F*_o_^2^) + (0.0112*P*)^2^ + 5.1398*P*] where *P* = (*F*_o_^2^ + 2*F*_c_^2^)/3
2268 reflections	(Δ/σ)_max_ = 0.001
84 parameters	Δρ_max_ = 1.31 e Å^−^^3^
0 restraints	Δρ_min_ = −1.16 e Å^−^^3^

### Bis(2-methylpyridine)gold(I) dibromidoaurate(I) (1) Fractional atomic coordinates and isotropic or equivalent isotropic displacement parameters (Å^2^)

**Table:**

	*x*	*y*	*z*	*U*_iso_*/*U*_eq_	
Au1	0.000000	0.41975 (3)	0.750000	0.01280 (5)	
Au2	0.000000	0.91951 (4)	0.750000	0.01467 (6)	
Br1	0.04466 (3)	0.92135 (7)	0.91263 (3)	0.02145 (9)	
N11	0.1128 (2)	0.4215 (5)	0.7285 (2)	0.0144 (6)	
C12	0.1984 (3)	0.4283 (6)	0.7993 (3)	0.0166 (8)	
C13	0.2741 (3)	0.4327 (6)	0.7815 (3)	0.0186 (8)	
H13	0.334013	0.436936	0.831171	0.022*	
C14	0.2616 (3)	0.4307 (7)	0.6915 (3)	0.0235 (9)	
H14	0.312952	0.434924	0.678944	0.028*	
C15	0.1743 (3)	0.4227 (7)	0.6193 (3)	0.0225 (9)	
H15	0.164670	0.421694	0.556923	0.027*	
C16	0.1012 (3)	0.4161 (7)	0.6405 (3)	0.0192 (8)	
H16	0.040936	0.407493	0.591510	0.023*	
C17	0.2067 (3)	0.4329 (7)	0.8949 (3)	0.0231 (9)	
H17A	0.172327	0.552494	0.901163	0.035*	
H17B	0.271147	0.446328	0.939583	0.035*	
H17C	0.181879	0.302807	0.906718	0.035*	

### Bis(2-methylpyridine)gold(I) dibromidoaurate(I) (1) Atomic displacement parameters (Å^2^)

**Table:**

	*U*^11^	*U*^22^	*U*^33^	*U*^12^	*U*^13^	*U*^23^
Au1	0.00881 (9)	0.01696 (11)	0.01346 (10)	0.000	0.00573 (7)	0.000
Au2	0.00994 (10)	0.01992 (11)	0.01375 (10)	0.000	0.00495 (8)	0.000
Br1	0.01998 (19)	0.0288 (2)	0.01402 (17)	0.00081 (19)	0.00620 (15)	0.00011 (18)
N11	0.0125 (15)	0.0139 (15)	0.0179 (16)	0.0000 (13)	0.0077 (13)	−0.0005 (14)
C12	0.0160 (19)	0.0088 (17)	0.023 (2)	−0.0010 (16)	0.0072 (16)	0.0003 (16)
C13	0.0133 (18)	0.0096 (18)	0.031 (2)	−0.0012 (15)	0.0085 (17)	0.0010 (17)
C14	0.022 (2)	0.015 (2)	0.045 (3)	0.0013 (18)	0.025 (2)	−0.0025 (19)
C15	0.031 (2)	0.017 (2)	0.029 (2)	−0.0031 (19)	0.022 (2)	−0.0035 (18)
C16	0.020 (2)	0.019 (2)	0.021 (2)	−0.0034 (18)	0.0108 (17)	−0.0016 (17)
C17	0.019 (2)	0.027 (2)	0.018 (2)	0.0001 (19)	0.0038 (17)	0.0007 (18)

### Bis(2-methylpyridine)gold(I) dibromidoaurate(I) (1) Geometric parameters (Å, º)

**Table:**

Au1—N11	2.027 (3)	C13—C14	1.378 (6)
Au1—N11^i^	2.027 (3)	C13—H13	0.9500
Au1—Au2	3.1907 (4)	C14—C15	1.383 (6)
Au1—Au2^ii^	3.1937 (4)	C14—H14	0.9500
Au2—Br1	2.3951 (4)	C15—C16	1.385 (6)
Au2—Br1^i^	2.3951 (4)	C15—H15	0.9500
N11—C16	1.350 (5)	C16—H16	0.9500
N11—C12	1.356 (5)	C17—H17A	0.9800
C12—C13	1.392 (6)	C17—H17B	0.9800
C12—C17	1.492 (6)	C17—H17C	0.9800
			
N11—Au1—N11^i^	179.35 (19)	C14—C13—C12	119.7 (4)
N11—Au1—Au2	89.67 (9)	C14—C13—H13	120.2
N11^i^—Au1—Au2	89.67 (9)	C12—C13—H13	120.2
N11—Au1—Au2^ii^	90.33 (9)	C13—C14—C15	120.0 (4)
N11^i^—Au1—Au2^ii^	90.33 (9)	C13—C14—H14	120.0
Au2—Au1—Au2^ii^	180.0	C15—C14—H14	120.0
Br1—Au2—Br1^i^	179.44 (2)	C14—C15—C16	118.2 (4)
Br1—Au2—Au1	90.282 (12)	C14—C15—H15	120.9
Br1^i^—Au2—Au1	90.282 (12)	C16—C15—H15	120.9
Br1—Au2—Au1^iii^	89.718 (12)	N11—C16—C15	122.1 (4)
Br1^i^—Au2—Au1^iii^	89.718 (12)	N11—C16—H16	119.0
Au1—Au2—Au1^iii^	180.0	C15—C16—H16	119.0
C16—N11—C12	119.7 (3)	C12—C17—H17A	109.5
C16—N11—Au1	118.2 (3)	C12—C17—H17B	109.5
C12—N11—Au1	122.1 (3)	H17A—C17—H17B	109.5
N11—C12—C13	120.3 (4)	C12—C17—H17C	109.5
N11—C12—C17	117.1 (4)	H17A—C17—H17C	109.5
C13—C12—C17	122.7 (4)	H17B—C17—H17C	109.5
			
N11^i^—Au1—Au2—Br1	70.36 (9)	C17—C12—C13—C14	−179.2 (4)
N11—Au1—Au2—Br1	−109.64 (9)	C12—C13—C14—C15	−0.6 (6)
C16—N11—C12—C13	0.9 (6)	C13—C14—C15—C16	−0.2 (6)
Au1—N11—C12—C13	−179.0 (3)	C12—N11—C16—C15	−1.7 (6)
C16—N11—C12—C17	−179.7 (4)	Au1—N11—C16—C15	178.2 (3)
Au1—N11—C12—C17	0.5 (5)	C14—C15—C16—N11	1.3 (6)
N11—C12—C13—C14	0.2 (6)		

Symmetry codes: (i) −*x*, *y*, −*z*+3/2; (ii) *x*, *y*−1, *z*; (iii) *x*, *y*+1, *z*.

### Bis(2-methylpyridine)gold(I) dibromidoaurate(I) (1) Hydrogen-bond geometry (Å, º)

**Table:**

*D*—H···*A*	*D*—H	H···*A*	*D*···*A*	*D*—H···*A*
C17—H17*B*···Br1^iv^	0.98	3.01	3.971 (4)	167
C14—H14···Au2^v^	0.95	2.75	3.581 (4)	147

Symmetry codes: (iv) −*x*+1/2, −*y*+3/2, −*z*+2; (v) *x*+1/2, *y*−1/2, *z*.

### Bis(3-methylpyridine)gold(I) dibromidoaurate(I) (2) Crystal data

**Table:**

[Au(C_6_H_7_N)_2_][AuBr_2_]	*F*(000) = 656
*M**_r_* = 740.00	*D*_x_ = 3.227 Mg m^−^^3^
Monoclinic, *C*2/*m*	Mo *K*α radiation, λ = 0.71073 Å
*a* = 16.7380 (6) Å	Cell parameters from 11080 reflections
*b* = 6.44097 (13) Å	θ = 2.8–30.7°
*c* = 8.1923 (3) Å	µ = 24.47 mm^−^^1^
β = 120.415 (5)°	*T* = 100 K
*V* = 761.65 (5) Å^3^	Block, colourless
*Z* = 2	0.15 × 0.15 × 0.10 mm

### Bis(3-methylpyridine)gold(I) dibromidoaurate(I) (2) Data collection

**Table:**

Oxford Diffraction Xcalibur, Eos diffractometer	1255 independent reflections
Radiation source: Enhance (Mo) X-ray Source	1162 reflections with *I* > 2σ(*I*)
Detector resolution: 16.1419 pixels mm^-1^	*R*_int_ = 0.034
ω scan	θ_max_ = 30.9°, θ_min_ = 2.8°
Absorption correction: multi-scan (CrysAlisPro; Rigaku OD, 2014)	*h* = −23→23
*T*_min_ = 0.487, *T*_max_ = 1.000	*k* = −9→9
25258 measured reflections	*l* = −11→11

### Bis(3-methylpyridine)gold(I) dibromidoaurate(I) (2) Refinement

**Table:**

Refinement on *F*^2^	Secondary atom site location: difference Fourier map
Least-squares matrix: full	Hydrogen site location: mixed
*R*[*F*^2^ > 2σ(*F*^2^)] = 0.013	H atoms treated by a mixture of independent and constrained refinement
*wR*(*F*^2^) = 0.029	*w* = 1/[σ^2^(*F*_o_^2^) + (0.0135*P*)^2^ + 1.2949*P*] where *P* = (*F*_o_^2^ + 2*F*_c_^2^)/3
*S* = 1.12	(Δ/σ)_max_ < 0.001
1255 reflections	Δρ_max_ = 1.02 e Å^−^^3^
65 parameters	Δρ_min_ = −0.93 e Å^−^^3^
1 restraint	Extinction correction: *SHELXL2019/3* (Sheldrick, 2015), *F*_c_^*^ = *kF*_c_[1 + 0.001*xF*_c_^2^λ^3^/sin(2θ)]^-1/4^
Primary atom site location: structure-invariant direct methods	Extinction coefficient: 0.00089 (6)

### Bis(3-methylpyridine)gold(I) dibromidoaurate(I) (2) Fractional atomic coordinates and isotropic or equivalent isotropic displacement parameters (Å^2^)

**Table:**

	*x*	*y*	*z*	*U*_iso_*/*U*_eq_	
Au1	0.000000	0.000000	0.000000	0.01689 (5)	
Au2	0.000000	0.500000	0.000000	0.01495 (5)	
Br1	0.04670 (2)	0.500000	0.32827 (5)	0.02509 (8)	
N11	0.11729 (18)	0.000000	−0.0135 (4)	0.0162 (5)	
C12	0.1127 (2)	0.000000	−0.1827 (4)	0.0169 (6)	
H12	0.053400	0.000000	−0.294313	0.020*	
C13	0.1909 (2)	0.000000	−0.2013 (4)	0.0162 (6)	
C14	0.2766 (2)	0.000000	−0.0343 (5)	0.0172 (6)	
H14	0.331892	0.000000	−0.040186	0.021*	
C15	0.2820 (2)	0.000000	0.1388 (5)	0.0192 (6)	
H15	0.340530	0.000000	0.252323	0.023*	
C16	0.2012 (2)	0.000000	0.1456 (5)	0.0185 (6)	
H16	0.204838	0.000000	0.265147	0.022*	
C17	0.1818 (2)	0.000000	−0.3930 (5)	0.0236 (7)	
H17A	0.153 (2)	0.125 (5)	−0.465 (5)	0.052 (10)*	
H17B	0.243 (3)	0.000000	−0.380 (8)	0.059 (16)*	

### Bis(3-methylpyridine)gold(I) dibromidoaurate(I) (2) Atomic displacement parameters (Å^2^)

**Table:**

	*U*^11^	*U*^22^	*U*^33^	*U*^12^	*U*^13^	*U*^23^
Au1	0.01603 (8)	0.01599 (8)	0.02303 (9)	0.000	0.01310 (7)	0.000
Au2	0.01066 (8)	0.01903 (8)	0.01457 (8)	0.000	0.00595 (6)	0.000
Br1	0.02468 (16)	0.03297 (18)	0.01477 (14)	0.000	0.00789 (12)	0.000
N11	0.0160 (12)	0.0143 (11)	0.0207 (12)	0.000	0.0110 (10)	0.000
C12	0.0135 (13)	0.0168 (14)	0.0181 (14)	0.000	0.0062 (11)	0.000
C13	0.0167 (14)	0.0132 (13)	0.0181 (14)	0.000	0.0084 (12)	0.000
C14	0.0122 (13)	0.0142 (13)	0.0239 (15)	0.000	0.0082 (12)	0.000
C15	0.0167 (14)	0.0165 (14)	0.0183 (14)	0.000	0.0043 (12)	0.000
C16	0.0233 (16)	0.0149 (14)	0.0190 (14)	0.000	0.0118 (13)	0.000
C17	0.0233 (17)	0.0281 (17)	0.0212 (16)	0.000	0.0126 (14)	0.000

### Bis(3-methylpyridine)gold(I) dibromidoaurate(I) (2) Geometric parameters (Å, º)

**Table:**

Au1—N11	2.021 (3)	C13—C14	1.394 (4)
Au1—N11^i^	2.021 (3)	C13—C17	1.499 (5)
Au1—Au2	3.2205 (1)	C14—C15	1.375 (5)
Au1—Au2^ii^	3.2205 (1)	C14—H14	0.9500
Au2—Br1^iii^	2.3906 (3)	C15—C16	1.381 (5)
Au2—Br1	2.3906 (3)	C15—H15	0.9500
N11—C16	1.348 (4)	C16—H16	0.9500
N11—C12	1.349 (4)	C17—H17A	0.97 (3)
C12—C13	1.391 (4)	C17—H17B	0.98 (3)
C12—H12	0.9500	C17—H17A^iv^	0.97 (3)
			
N11—Au1—N11^i^	180.0	C12—C13—C14	116.8 (3)
N11—Au1—Au2	90.0	C12—C13—C17	120.8 (3)
N11^i^—Au1—Au2	90.0	C14—C13—C17	122.4 (3)
N11—Au1—Au2^ii^	90.0	C15—C14—C13	120.6 (3)
N11^i^—Au1—Au2^ii^	90.0	C15—C14—H14	119.7
Au2—Au1—Au2^ii^	180.0	C13—C14—H14	119.7
Br1^iii^—Au2—Br1	180.0	C14—C15—C16	119.2 (3)
Br1^iii^—Au2—Au1	90.0	C14—C15—H15	120.4
Br1—Au2—Au1	90.0	C16—C15—H15	120.4
Br1^iii^—Au2—Au1^v^	90.0	N11—C16—C15	121.5 (3)
Br1—Au2—Au1^v^	90.0	N11—C16—H16	119.2
Au1—Au2—Au1^v^	180.0	C15—C16—H16	119.2
C16—N11—C12	118.9 (3)	C13—C17—H17A	113 (2)
C16—N11—Au1	120.8 (2)	C13—C17—H17B	110 (3)
C12—N11—Au1	120.3 (2)	H17A—C17—H17B	104 (3)
N11—C12—C13	123.0 (3)	C13—C17—H17A^iv^	113 (2)
N11—C12—H12	118.5	H17A—C17—H17A^iv^	112 (4)
C13—C12—H12	118.5	H17B—C17—H17A^iv^	104 (3)
			
N11—Au1—Au2—Br1	−106.73 (8)	C12—C13—C14—C15	0.000 (1)
N11^i^—Au1—Au2—Br1	73.27 (8)	C17—C13—C14—C15	180.000 (1)
C16—N11—C12—C13	0.000 (1)	C13—C14—C15—C16	0.000 (1)
Au1—N11—C12—C13	180.000 (1)	C12—N11—C16—C15	0.000 (1)
N11—C12—C13—C14	0.000 (1)	Au1—N11—C16—C15	180.000 (1)
N11—C12—C13—C17	180.000 (1)	C14—C15—C16—N11	0.000 (1)

Symmetry codes: (i) −*x*, −*y*, −*z*; (ii) *x*, *y*−1, *z*; (iii) −*x*, −*y*+1, −*z*; (iv) *x*, −*y*, *z*; (v) *x*, *y*+1, *z*.

### Bis(3-methylpyridine)gold(I) dibromidoaurate(I) (2) Hydrogen-bond geometry (Å, º)

**Table:**

*D*—H···*A*	*D*—H	H···*A*	*D*···*A*	*D*—H···*A*
C15—H15···Br1^vi^	0.95	2.97	3.822 (3)	151
C14—H14···Au2^vii^	0.95	2.66	3.604 (3)	171

Symmetry codes: (vi) −*x*+1/2, −*y*+1/2, −*z*+1; (vii) *x*+1/2, *y*−1/2, *z*.

### Bis(3,5-dimethylpyridine)gold(I) dichloridoaurate(I) (3) Crystal data

**Table:**

[Au(C_7_H_9_N)_2_][AuCl_2_]	*Z* = 2
*M**_r_* = 679.14	*F*(000) = 616
Triclinic, *P*1	*D*_x_ = 2.689 Mg m^−^^3^
*a* = 6.7718 (3) Å	Mo *K*α radiation, λ = 0.71073 Å
*b* = 8.5627 (5) Å	Cell parameters from 11878 reflections
*c* = 15.1064 (8) Å	θ = 2.8–29.9°
α = 105.356 (5)°	µ = 17.78 mm^−^^1^
β = 90.788 (4)°	*T* = 100 K
γ = 96.311 (4)°	Plate, colourless
*V* = 838.71 (8) Å^3^	0.15 × 0.15 × 0.01 mm

### Bis(3,5-dimethylpyridine)gold(I) dichloridoaurate(I) (3) Data collection

**Table:**

Oxford Diffraction Xcalibur, Eos diffractometer	5018 independent reflections
Radiation source: Enhance (Mo) X-ray Source	4253 reflections with *I* > 2σ(*I*)
Graphite monochromator	*R*_int_ = 0.054
Detector resolution: 16.1419 pixels mm^-1^	θ_max_ = 31.0°, θ_min_ = 2.5°
ω scan	*h* = −9→9
Absorption correction: multi-scan (CrysAlisPro; Rigaku OD, 2014)	*k* = −11→12
*T*_min_ = 0.330, *T*_max_ = 1.000	*l* = −21→21
44852 measured reflections	

### Bis(3,5-dimethylpyridine)gold(I) dichloridoaurate(I) (3) Refinement

**Table:**

Refinement on *F*^2^	Secondary atom site location: difference Fourier map
Least-squares matrix: full	Hydrogen site location: inferred from neighbouring sites
*R*[*F*^2^ > 2σ(*F*^2^)] = 0.022	H-atom parameters constrained
*wR*(*F*^2^) = 0.040	*w* = 1/[σ^2^(*F*_o_^2^) + (0.0126*P*)^2^] where *P* = (*F*_o_^2^ + 2*F*_c_^2^)/3
*S* = 1.06	(Δ/σ)_max_ = 0.001
5018 reflections	Δρ_max_ = 1.04 e Å^−^^3^
189 parameters	Δρ_min_ = −0.87 e Å^−^^3^
0 restraints	Extinction correction: *SHELXL2019/3* (Sheldrick, 2015), *F*_c_^*^ = *kF*_c_[1 + 0.001*xF*_c_^2^λ^3^/sin(2θ)]^-1/4^
Primary atom site location: structure-invariant direct methods	Extinction coefficient: 0.00055 (6)

### Bis(3,5-dimethylpyridine)gold(I) dichloridoaurate(I) (3) Fractional atomic coordinates and isotropic or equivalent isotropic displacement parameters (Å^2^)

**Table:**

	*x*	*y*	*z*	*U*_iso_*/*U*_eq_	
Au1	0.19404 (2)	0.41613 (2)	0.44756 (2)	0.01391 (4)	
Au2	0.500000	1.000000	1.000000	0.02024 (5)	
Au3	0.000000	1.000000	0.500000	0.01968 (5)	
Cl1	0.52929 (15)	0.75061 (11)	1.01852 (7)	0.0298 (2)	
Cl2	0.16983 (15)	0.81866 (10)	0.40299 (6)	0.0265 (2)	
N11	0.2983 (4)	0.5264 (3)	0.57698 (19)	0.0143 (6)	
C12	0.3289 (5)	0.6906 (4)	0.6049 (2)	0.0154 (7)	
H12	0.302272	0.750675	0.562391	0.019*	
C13	0.3978 (5)	0.7752 (4)	0.6935 (2)	0.0144 (7)	
C14	0.4386 (4)	0.6835 (4)	0.7535 (2)	0.0138 (7)	
H14	0.485411	0.737610	0.814703	0.017*	
C15	0.4122 (5)	0.5140 (4)	0.7256 (2)	0.0163 (7)	
C16	0.3408 (5)	0.4399 (4)	0.6355 (2)	0.0140 (7)	
H16	0.321583	0.324214	0.614935	0.017*	
C17	0.4251 (5)	0.9581 (4)	0.7204 (2)	0.0187 (7)	
H17A	0.546670	0.997066	0.694511	0.028*	
H17B	0.435787	0.998634	0.787560	0.028*	
H17C	0.310597	0.997990	0.696811	0.028*	
C18	0.4577 (5)	0.4110 (4)	0.7885 (2)	0.0217 (8)	
H18A	0.559862	0.341875	0.762129	0.033*	
H18B	0.336588	0.342098	0.795489	0.033*	
H18C	0.506095	0.481966	0.848735	0.033*	
N21	0.1074 (4)	0.3082 (3)	0.31525 (19)	0.0131 (6)	
C22	0.0795 (5)	0.3988 (4)	0.2563 (2)	0.0154 (7)	
H22	0.088903	0.514002	0.279473	0.018*	
C23	0.0379 (5)	0.3303 (4)	0.1639 (2)	0.0148 (7)	
C24	0.0261 (5)	0.1609 (4)	0.1313 (2)	0.0165 (7)	
H24	−0.001606	0.110207	0.067693	0.020*	
C25	0.0543 (5)	0.0655 (4)	0.1908 (2)	0.0158 (7)	
C26	0.0945 (5)	0.1446 (4)	0.2825 (2)	0.0141 (7)	
H26	0.113969	0.081270	0.324199	0.017*	
C27	0.0083 (6)	0.4329 (4)	0.0993 (3)	0.0236 (8)	
H27A	0.010261	0.547109	0.134369	0.035*	
H27B	−0.120006	0.395214	0.065353	0.035*	
H27C	0.115449	0.423334	0.055848	0.035*	
C28	0.0428 (5)	−0.1171 (4)	0.1578 (2)	0.0194 (7)	
H28A	0.154670	−0.147117	0.118872	0.029*	
H28B	−0.082559	−0.160796	0.122245	0.029*	
H28C	0.048879	−0.162509	0.210764	0.029*	

### Bis(3,5-dimethylpyridine)gold(I) dichloridoaurate(I) (3) Atomic displacement parameters (Å^2^)

**Table:**

	*U*^11^	*U*^22^	*U*^33^	*U*^12^	*U*^13^	*U*^23^
Au1	0.01280 (7)	0.01698 (7)	0.00988 (7)	0.00158 (5)	−0.00054 (5)	0.00011 (5)
Au2	0.02307 (11)	0.02408 (10)	0.01180 (10)	0.00159 (8)	−0.00070 (8)	0.00230 (8)
Au3	0.02985 (11)	0.01651 (9)	0.01332 (10)	0.00124 (8)	−0.00102 (8)	0.00583 (7)
Cl1	0.0419 (6)	0.0270 (5)	0.0199 (5)	0.0048 (4)	−0.0029 (4)	0.0054 (4)
Cl2	0.0420 (6)	0.0198 (4)	0.0194 (5)	0.0065 (4)	0.0042 (4)	0.0069 (4)
N11	0.0104 (13)	0.0185 (14)	0.0129 (15)	0.0013 (11)	−0.0003 (11)	0.0024 (11)
C12	0.0146 (16)	0.0169 (16)	0.0153 (18)	0.0040 (13)	0.0017 (13)	0.0042 (13)
C13	0.0110 (16)	0.0163 (16)	0.0141 (17)	0.0016 (12)	0.0038 (13)	0.0010 (13)
C14	0.0104 (15)	0.0188 (16)	0.0095 (16)	−0.0017 (12)	0.0004 (12)	0.0005 (13)
C15	0.0146 (17)	0.0194 (17)	0.0154 (18)	0.0014 (13)	0.0009 (13)	0.0057 (14)
C16	0.0138 (16)	0.0127 (15)	0.0146 (17)	0.0017 (12)	0.0017 (13)	0.0018 (13)
C17	0.0182 (18)	0.0154 (16)	0.0221 (19)	0.0014 (13)	0.0004 (15)	0.0046 (14)
C18	0.031 (2)	0.0199 (18)	0.0147 (18)	0.0016 (15)	−0.0010 (15)	0.0063 (14)
N21	0.0099 (13)	0.0146 (13)	0.0134 (15)	0.0012 (10)	0.0015 (11)	0.0016 (11)
C22	0.0144 (16)	0.0121 (15)	0.0188 (18)	0.0023 (12)	0.0003 (14)	0.0024 (13)
C23	0.0129 (16)	0.0129 (15)	0.0184 (18)	0.0034 (12)	0.0015 (13)	0.0032 (13)
C24	0.0214 (18)	0.0169 (16)	0.0100 (17)	0.0030 (13)	0.0005 (14)	0.0010 (13)
C25	0.0170 (17)	0.0134 (15)	0.0170 (18)	0.0020 (13)	0.0006 (14)	0.0039 (13)
C26	0.0119 (16)	0.0147 (15)	0.0166 (18)	0.0029 (12)	0.0029 (13)	0.0051 (13)
C27	0.034 (2)	0.0177 (17)	0.019 (2)	0.0032 (15)	−0.0024 (16)	0.0063 (15)
C28	0.0270 (19)	0.0133 (16)	0.0169 (19)	0.0035 (14)	0.0026 (15)	0.0018 (14)

### Bis(3,5-dimethylpyridine)gold(I) dichloridoaurate(I) (3) Geometric parameters (Å, º)

**Table:**

Au1—N11	2.013 (3)	C18—H18A	0.9800
Au1—N21	2.016 (3)	C18—H18B	0.9800
Au1—Au1^i^	3.3495 (3)	C18—H18C	0.9800
Au2—Cl1	2.2551 (9)	N21—C26	1.349 (4)
Au2—Cl1^ii^	2.2551 (9)	N21—C22	1.351 (4)
Au3—Cl2	2.2617 (9)	C22—C23	1.375 (5)
Au3—Cl2^iii^	2.2617 (9)	C22—H22	0.9500
N11—C16	1.342 (4)	C23—C24	1.396 (4)
N11—C12	1.348 (4)	C23—C27	1.500 (5)
C12—C13	1.388 (4)	C24—C25	1.389 (4)
C12—H12	0.9500	C24—H24	0.9500
C13—C14	1.390 (5)	C25—C26	1.380 (5)
C13—C17	1.500 (4)	C25—C28	1.503 (4)
C14—C15	1.390 (4)	C26—H26	0.9500
C14—H14	0.9500	C27—H27A	0.9800
C15—C16	1.394 (4)	C27—H27B	0.9800
C15—C18	1.508 (4)	C27—H27C	0.9800
C16—H16	0.9500	C28—H28A	0.9800
C17—H17A	0.9800	C28—H28B	0.9800
C17—H17B	0.9800	C28—H28C	0.9800
C17—H17C	0.9800		
			
N11—Au1—N21	176.33 (10)	H18A—C18—H18C	109.5
N11—Au1—Au1^i^	75.62 (7)	H18B—C18—H18C	109.5
N21—Au1—Au1^i^	107.56 (7)	C26—N21—C22	119.0 (3)
Cl1—Au2—Cl1^ii^	180.0	C26—N21—Au1	120.2 (2)
Cl2—Au3—Cl2^iii^	180.0	C22—N21—Au1	120.6 (2)
C16—N11—C12	119.4 (3)	N21—C22—C23	122.4 (3)
C16—N11—Au1	121.4 (2)	N21—C22—H22	118.8
C12—N11—Au1	119.2 (2)	C23—C22—H22	118.8
N11—C12—C13	122.5 (3)	C22—C23—C24	117.8 (3)
N11—C12—H12	118.7	C22—C23—C27	121.7 (3)
C13—C12—H12	118.7	C24—C23—C27	120.6 (3)
C12—C13—C14	117.3 (3)	C25—C24—C23	120.8 (3)
C12—C13—C17	119.7 (3)	C25—C24—H24	119.6
C14—C13—C17	123.0 (3)	C23—C24—H24	119.6
C15—C14—C13	121.2 (3)	C26—C25—C24	117.5 (3)
C15—C14—H14	119.4	C26—C25—C28	120.4 (3)
C13—C14—H14	119.4	C24—C25—C28	122.0 (3)
C14—C15—C16	117.4 (3)	N21—C26—C25	122.6 (3)
C14—C15—C18	122.5 (3)	N21—C26—H26	118.7
C16—C15—C18	120.1 (3)	C25—C26—H26	118.7
N11—C16—C15	122.3 (3)	C23—C27—H27A	109.5
N11—C16—H16	118.9	C23—C27—H27B	109.5
C15—C16—H16	118.9	H27A—C27—H27B	109.5
C13—C17—H17A	109.5	C23—C27—H27C	109.5
C13—C17—H17B	109.5	H27A—C27—H27C	109.5
H17A—C17—H17B	109.5	H27B—C27—H27C	109.5
C13—C17—H17C	109.5	C25—C28—H28A	109.5
H17A—C17—H17C	109.5	C25—C28—H28B	109.5
H17B—C17—H17C	109.5	H28A—C28—H28B	109.5
C15—C18—H18A	109.5	C25—C28—H28C	109.5
C15—C18—H18B	109.5	H28A—C28—H28C	109.5
H18A—C18—H18B	109.5	H28B—C28—H28C	109.5
C15—C18—H18C	109.5		
			
C16—N11—C12—C13	−1.9 (5)	C26—N21—C22—C23	0.1 (5)
Au1—N11—C12—C13	179.1 (2)	Au1—N21—C22—C23	174.0 (2)
N11—C12—C13—C14	1.1 (5)	N21—C22—C23—C24	−0.4 (5)
N11—C12—C13—C17	−178.9 (3)	N21—C22—C23—C27	−179.7 (3)
C12—C13—C14—C15	0.3 (5)	C22—C23—C24—C25	0.4 (5)
C17—C13—C14—C15	−179.7 (3)	C27—C23—C24—C25	179.7 (3)
C13—C14—C15—C16	−0.8 (5)	C23—C24—C25—C26	−0.1 (5)
C13—C14—C15—C18	179.1 (3)	C23—C24—C25—C28	−179.9 (3)
C12—N11—C16—C15	1.3 (5)	C22—N21—C26—C25	0.1 (5)
Au1—N11—C16—C15	−179.7 (2)	Au1—N21—C26—C25	−173.8 (2)
C14—C15—C16—N11	0.0 (5)	C24—C25—C26—N21	−0.1 (5)
C18—C15—C16—N11	−180.0 (3)	C28—C25—C26—N21	179.6 (3)

Symmetry codes: (i) −*x*, −*y*+1, −*z*+1; (ii) −*x*+1, −*y*+2, −*z*+2; (iii) −*x*, −*y*+2, −*z*+1.

### Bis(3,5-dimethylpyridine)gold(I) dichloridoaurate(I) (3) Hydrogen-bond geometry (Å, º)

**Table:**

*D*—H···*A*	*D*—H	H···*A*	*D*···*A*	*D*—H···*A*
C18—H18*C*···Cl1	0.98	2.95	3.881 (4)	160
C28—H28*B*···Cl1^iv^	0.98	2.96	3.934 (4)	173
C12—H12···Cl2	0.95	2.78	3.689 (3)	160
C22—H22···Cl2	0.95	2.77	3.670 (3)	159
C26—H26···Cl2^v^	0.95	2.86	3.787 (3)	164
C26—H26···Au3^v^	0.95	3.01	3.844 (3)	148
C26—H26···Au3^i^	0.95	3.01	3.844 (3)	148
C17—H17*A*···Cl2^vi^	0.98	3.00	3.941 (4)	162

Symmetry codes: (i) −*x*, −*y*+1, −*z*+1; (iv) *x*−1, *y*−1, *z*−1; (v) *x*, *y*−1, *z*; (vi) −*x*+1, −*y*+2, −*z*+1.

### Bis(3,5-dimethylpyridine)gold(I) dibromidoaurate(I) (4) Crystal data

**Table:**

[Au(C_7_H_9_N)_2_][AuBr_2_]	*Z* = 2
*M**_r_* = 768.06	*F*(000) = 688
Triclinic, *P*1	*D*_x_ = 2.938 Mg m^−^^3^
*a* = 6.8343 (2) Å	Mo *K*α radiation, λ = 0.71073 Å
*b* = 8.6676 (3) Å	Cell parameters from 11289 reflections
*c* = 15.4049 (6) Å	θ = 2.8–30.4°
α = 105.720 (3)°	µ = 21.48 mm^−^^1^
β = 90.741 (3)°	*T* = 100 K
γ = 98.242 (3)°	Block, colourless
*V* = 868.05 (6) Å^3^	0.15 × 0.10 × 0.03 mm

### Bis(3,5-dimethylpyridine)gold(I) dibromidoaurate(I) (4) Data collection

**Table:**

Oxford Diffraction Xcalibur, Eos diffractometer	5187 independent reflections
Radiation source: Enhance (Mo) X-ray Source	4424 reflections with *I* > 2σ(*I*)
Graphite monochromator	*R*_int_ = 0.055
Detector resolution: 16.1419 pixels mm^-1^	θ_max_ = 30.9°, θ_min_ = 2.5°
ω scan	*h* = −9→9
Absorption correction: multi-scan (CrysAlisPro; Rigaku OD, 2014)	*k* = −12→12
*T*_min_ = 0.256, *T*_max_ = 1.000	*l* = −22→21
47547 measured reflections	

### Bis(3,5-dimethylpyridine)gold(I) dibromidoaurate(I) (4) Refinement

**Table:**

Refinement on *F*^2^	Primary atom site location: structure-invariant direct methods
Least-squares matrix: full	Secondary atom site location: difference Fourier map
*R*[*F*^2^ > 2σ(*F*^2^)] = 0.026	Hydrogen site location: inferred from neighbouring sites
*wR*(*F*^2^) = 0.054	H-atom parameters constrained
*S* = 1.05	*w* = 1/[σ^2^(*F*_o_^2^) + (0.0219*P*)^2^ + 1.2753*P*] where *P* = (*F*_o_^2^ + 2*F*_c_^2^)/3
5187 reflections	(Δ/σ)_max_ = 0.001
188 parameters	Δρ_max_ = 1.24 e Å^−^^3^
0 restraints	Δρ_min_ = −1.80 e Å^−^^3^

### Bis(3,5-dimethylpyridine)gold(I) dibromidoaurate(I) (4) Fractional atomic coordinates and isotropic or equivalent isotropic displacement parameters (Å^2^)

**Table:**

	*x*	*y*	*z*	*U*_iso_*/*U*_eq_	
Au1	0.19570 (2)	0.41886 (2)	0.44593 (2)	0.01603 (5)	
Au2	0.500000	1.000000	1.000000	0.01924 (6)	
Au3	0.000000	1.000000	0.500000	0.02067 (6)	
Br1	0.52006 (7)	0.73692 (6)	1.01523 (3)	0.02540 (10)	
Br2	0.20599 (7)	0.83275 (5)	0.40665 (3)	0.02658 (10)	
N11	0.2943 (5)	0.5230 (4)	0.5751 (2)	0.0152 (7)	
C12	0.3267 (6)	0.6861 (5)	0.6073 (3)	0.0155 (8)	
H12	0.303987	0.749753	0.567858	0.019*	
C13	0.3920 (5)	0.7640 (5)	0.6962 (3)	0.0140 (8)	
C14	0.4273 (6)	0.6685 (5)	0.7522 (3)	0.0153 (8)	
H14	0.471316	0.718623	0.813470	0.018*	
C15	0.3989 (6)	0.4995 (5)	0.7196 (3)	0.0160 (8)	
C16	0.3306 (6)	0.4319 (5)	0.6305 (3)	0.0174 (8)	
H16	0.308315	0.317109	0.607344	0.021*	
C17	0.4223 (6)	0.9468 (5)	0.7279 (3)	0.0208 (9)	
H17A	0.550060	0.989932	0.708878	0.031*	
H17B	0.420962	0.982022	0.793930	0.031*	
H17C	0.315707	0.987083	0.701549	0.031*	
C18	0.4381 (7)	0.3933 (5)	0.7785 (3)	0.0228 (9)	
H18A	0.315004	0.323694	0.783605	0.034*	
H18B	0.487960	0.461321	0.838657	0.034*	
H18C	0.537008	0.325742	0.751645	0.034*	
N21	0.1143 (5)	0.3142 (4)	0.3145 (2)	0.0148 (7)	
C22	0.0880 (6)	0.4055 (5)	0.2581 (3)	0.0162 (8)	
H22	0.096229	0.519517	0.282510	0.019*	
C23	0.0492 (6)	0.3390 (5)	0.1660 (3)	0.0164 (8)	
C24	0.0346 (6)	0.1713 (5)	0.1324 (3)	0.0169 (8)	
H24	0.008230	0.121915	0.069543	0.020*	
C25	0.0581 (6)	0.0759 (5)	0.1894 (3)	0.0170 (8)	
C26	0.0981 (6)	0.1531 (5)	0.2809 (3)	0.0155 (8)	
H26	0.114579	0.089239	0.321041	0.019*	
C27	0.0228 (7)	0.4448 (5)	0.1048 (3)	0.0236 (10)	
H27A	0.031442	0.557834	0.140983	0.035*	
H27B	−0.107061	0.409300	0.072230	0.035*	
H27C	0.126917	0.436179	0.061299	0.035*	
C28	0.0451 (7)	−0.1053 (5)	0.1556 (3)	0.0224 (9)	
H28A	−0.083934	−0.151890	0.123778	0.034*	
H28B	0.060274	−0.150212	0.206816	0.034*	
H28C	0.150565	−0.132027	0.114132	0.034*	

### Bis(3,5-dimethylpyridine)gold(I) dibromidoaurate(I) (4) Atomic displacement parameters (Å^2^)

**Table:**

	*U*^11^	*U*^22^	*U*^33^	*U*^12^	*U*^13^	*U*^23^
Au1	0.01280 (7)	0.02041 (8)	0.01248 (8)	0.00253 (6)	−0.00055 (6)	0.00056 (6)
Au2	0.02099 (11)	0.02163 (12)	0.01407 (12)	0.00233 (9)	0.00009 (9)	0.00371 (9)
Au3	0.02961 (13)	0.01742 (11)	0.01480 (12)	0.00197 (9)	−0.00004 (9)	0.00501 (9)
Br1	0.0338 (2)	0.0229 (2)	0.0194 (2)	0.00489 (18)	−0.00102 (19)	0.00537 (18)
Br2	0.0392 (3)	0.0218 (2)	0.0208 (2)	0.00813 (19)	0.0053 (2)	0.00760 (18)
N11	0.0112 (15)	0.0183 (17)	0.0135 (18)	0.0009 (13)	0.0010 (13)	0.0006 (14)
C12	0.0118 (17)	0.019 (2)	0.017 (2)	0.0042 (15)	0.0026 (15)	0.0050 (16)
C13	0.0107 (17)	0.0152 (18)	0.016 (2)	0.0026 (14)	0.0022 (15)	0.0040 (16)
C14	0.0124 (17)	0.020 (2)	0.013 (2)	−0.0001 (15)	−0.0010 (15)	0.0042 (16)
C15	0.0136 (18)	0.0156 (19)	0.020 (2)	0.0033 (15)	0.0025 (16)	0.0053 (16)
C16	0.0135 (18)	0.0150 (19)	0.022 (2)	0.0007 (15)	0.0014 (16)	0.0032 (17)
C17	0.022 (2)	0.016 (2)	0.023 (2)	0.0023 (16)	0.0019 (18)	0.0029 (18)
C18	0.029 (2)	0.018 (2)	0.023 (2)	0.0042 (18)	0.0009 (19)	0.0082 (18)
N21	0.0092 (14)	0.0169 (16)	0.0163 (18)	0.0011 (12)	0.0026 (13)	0.0015 (14)
C22	0.0120 (17)	0.0173 (19)	0.019 (2)	0.0047 (15)	0.0017 (16)	0.0036 (16)
C23	0.0152 (18)	0.0160 (19)	0.018 (2)	0.0032 (15)	−0.0002 (16)	0.0051 (16)
C24	0.022 (2)	0.0155 (19)	0.010 (2)	0.0003 (16)	−0.0011 (16)	−0.0005 (16)
C25	0.0128 (18)	0.0152 (19)	0.022 (2)	0.0007 (15)	0.0010 (16)	0.0051 (17)
C26	0.0115 (17)	0.0174 (19)	0.018 (2)	0.0015 (15)	0.0027 (15)	0.0063 (17)
C27	0.035 (2)	0.020 (2)	0.018 (2)	0.0070 (19)	−0.0028 (19)	0.0062 (18)
C28	0.027 (2)	0.016 (2)	0.020 (2)	0.0005 (17)	0.0010 (18)	−0.0003 (17)

### Bis(3,5-dimethylpyridine)gold(I) dibromidoaurate(I) (4) Geometric parameters (Å, º)

**Table:**

Au1—N11	2.012 (3)	C18—H18A	0.9800
Au1—N21	2.016 (4)	C18—H18B	0.9800
Au1—Au1^i^	3.4400 (3)	C18—H18C	0.9800
Au2—Br1	2.3775 (5)	N21—C26	1.338 (5)
Au2—Br1^ii^	2.3775 (5)	N21—C22	1.350 (5)
Au3—Br2^iii^	2.3789 (5)	C22—C23	1.386 (6)
Au3—Br2	2.3789 (5)	C22—H22	0.9500
N11—C12	1.349 (5)	C23—C24	1.393 (6)
N11—C16	1.353 (6)	C23—C27	1.509 (6)
C12—C13	1.387 (6)	C24—C25	1.382 (6)
C12—H12	0.9500	C24—H24	0.9500
C13—C14	1.389 (6)	C25—C26	1.392 (6)
C13—C17	1.508 (6)	C25—C28	1.504 (6)
C14—C15	1.397 (6)	C26—H26	0.9500
C14—H14	0.9500	C27—H27A	0.9800
C15—C16	1.384 (6)	C27—H27B	0.9800
C15—C18	1.503 (6)	C27—H27C	0.9800
C16—H16	0.9500	C28—H28A	0.9800
C17—H17A	0.9800	C28—H28B	0.9800
C17—H17B	0.9800	C28—H28C	0.9800
C17—H17C	0.9800		
			
N11—Au1—N21	176.50 (13)	H18B—C18—H18C	109.5
Br1—Au2—Br1^ii^	180.0	C26—N21—C22	119.1 (4)
Br2^iii^—Au3—Br2	180.0	C26—N21—Au1	120.0 (3)
C12—N11—C16	119.1 (4)	C22—N21—Au1	120.7 (3)
C12—N11—Au1	119.9 (3)	N21—C22—C23	122.3 (4)
C16—N11—Au1	120.9 (3)	N21—C22—H22	118.8
N11—C12—C13	122.3 (4)	C23—C22—H22	118.8
N11—C12—H12	118.9	C22—C23—C24	117.5 (4)
C13—C12—H12	118.9	C22—C23—C27	121.0 (4)
C12—C13—C14	117.8 (4)	C24—C23—C27	121.5 (4)
C12—C13—C17	119.4 (4)	C25—C24—C23	120.8 (4)
C14—C13—C17	122.8 (4)	C25—C24—H24	119.6
C13—C14—C15	120.8 (4)	C23—C24—H24	119.6
C13—C14—H14	119.6	C24—C25—C26	117.8 (4)
C15—C14—H14	119.6	C24—C25—C28	122.4 (4)
C16—C15—C14	117.5 (4)	C26—C25—C28	119.9 (4)
C16—C15—C18	120.6 (4)	N21—C26—C25	122.4 (4)
C14—C15—C18	121.9 (4)	N21—C26—H26	118.8
N11—C16—C15	122.4 (4)	C25—C26—H26	118.8
N11—C16—H16	118.8	C23—C27—H27A	109.5
C15—C16—H16	118.8	C23—C27—H27B	109.5
C13—C17—H17A	109.5	H27A—C27—H27B	109.5
C13—C17—H17B	109.5	C23—C27—H27C	109.5
H17A—C17—H17B	109.5	H27A—C27—H27C	109.5
C13—C17—H17C	109.5	H27B—C27—H27C	109.5
H17A—C17—H17C	109.5	C25—C28—H28A	109.5
H17B—C17—H17C	109.5	C25—C28—H28B	109.5
C15—C18—H18A	109.5	H28A—C28—H28B	109.5
C15—C18—H18B	109.5	C25—C28—H28C	109.5
H18A—C18—H18B	109.5	H28A—C28—H28C	109.5
C15—C18—H18C	109.5	H28B—C28—H28C	109.5
H18A—C18—H18C	109.5		
			
C16—N11—C12—C13	−1.5 (6)	C26—N21—C22—C23	−1.6 (6)
Au1—N11—C12—C13	179.1 (3)	Au1—N21—C22—C23	174.2 (3)
N11—C12—C13—C14	1.1 (6)	N21—C22—C23—C24	1.1 (6)
N11—C12—C13—C17	−179.2 (4)	N21—C22—C23—C27	−179.3 (4)
C12—C13—C14—C15	0.4 (6)	C22—C23—C24—C25	0.0 (6)
C17—C13—C14—C15	−179.4 (4)	C27—C23—C24—C25	−179.6 (4)
C13—C14—C15—C16	−1.4 (6)	C23—C24—C25—C26	−0.5 (6)
C13—C14—C15—C18	179.3 (4)	C23—C24—C25—C28	−179.4 (4)
C12—N11—C16—C15	0.4 (6)	C22—N21—C26—C25	1.1 (6)
Au1—N11—C16—C15	179.8 (3)	Au1—N21—C26—C25	−174.7 (3)
C14—C15—C16—N11	1.0 (6)	C24—C25—C26—N21	0.0 (6)
C18—C15—C16—N11	−179.7 (4)	C28—C25—C26—N21	178.9 (4)

Symmetry codes: (i) −*x*, −*y*+1, −*z*+1; (ii) −*x*+1, −*y*+2, −*z*+2; (iii) −*x*, −*y*+2, −*z*+1.

### Bis(3,5-dimethylpyridine)gold(I) dibromidoaurate(I) (4) Hydrogen-bond geometry (Å, º)

**Table:**

*D*—H···*A*	*D*—H	H···*A*	*D*···*A*	*D*—H···*A*
C14—H14···Br1	0.95	3.08	3.961 (4)	155
C18—H18*B*···Br1	0.98	3.08	4.008 (5)	159
C27—H27*B*···Br1^i^	0.98	3.06	4.029 (5)	172
C27—H27*C*···Br1^iv^	0.98	3.10	3.955 (5)	147
C28—H28*A*···Br1^v^	0.98	3.05	4.026 (5)	173
C12—H12···Br2	0.95	2.86	3.771 (4)	160
C17—H17*A*···Br2^vi^	0.98	3.02	3.911 (4)	151
C22—H22···Br2	0.95	2.86	3.755 (4)	158
C26—H26···Br2^vii^	0.95	3.01	3.933 (4)	166

Symmetry codes: (i) −*x*, −*y*+1, −*z*+1; (iv) −*x*+1, −*y*+1, −*z*+1; (v) *x*−1, *y*−1, *z*−1; (vi) −*x*+1, −*y*+2, −*z*+1; (vii) *x*, *y*−1, *z*.

### 2-Bromopyridinium dibromidoaurate(I)–2-bromopyridine (1/1) (5) Crystal data

**Table:**

(C_5_H_5_BrN)[AuBr_2_]·C_5_H_4_BrN	*Z* = 2
*M**_r_* = 673.80	*F*(000) = 604
Triclinic, *P*1	*D*_x_ = 3.044 Mg m^−^^3^
*a* = 7.9931 (4) Å	Mo *K*α radiation, λ = 0.71073 Å
*b* = 8.4672 (3) Å	Cell parameters from 14102 reflections
*c* = 11.3923 (5) Å	θ = 2.4–30.6°
α = 87.202 (4)°	µ = 20.86 mm^−^^1^
β = 74.635 (4)°	*T* = 100 K
γ = 81.406 (4)°	Block, colourless
*V* = 735.09 (6) Å^3^	0.15 × 0.10 × 0.04 mm

### 2-Bromopyridinium dibromidoaurate(I)–2-bromopyridine (1/1) (5) Data collection

**Table:**

Oxford Diffrection Xcalibur, Eos diffractometer	4361 independent reflections
Radiation source: Enhance (Mo) X-ray Source	3917 reflections with *I* > 2σ(*I*)
Graphite monochromator	*R*_int_ = 0.044
Detector resolution: 16.1419 pixels mm^-1^	θ_max_ = 30.9°, θ_min_ = 2.4°
ω scan	*h* = −11→11
Absorption correction: multi-scan (CrysAlisPro; Rigaku OD, 2014)	*k* = −11→12
*T*_min_ = 0.241, *T*_max_ = 1.000	*l* = −16→16
42320 measured reflections	

### 2-Bromopyridinium dibromidoaurate(I)–2-bromopyridine (1/1) (5) Refinement

**Table:**

Refinement on *F*^2^	Primary atom site location: structure-invariant direct methods
Least-squares matrix: full	Secondary atom site location: difference Fourier map
*R*[*F*^2^ > 2σ(*F*^2^)] = 0.023	Hydrogen site location: mixed
*wR*(*F*^2^) = 0.048	H atoms treated by a mixture of independent and constrained refinement
*S* = 1.07	*w* = 1/[σ^2^(*F*_o_^2^) + (0.0232*P*)^2^ + 0.2423*P*] where *P* = (*F*_o_^2^ + 2*F*_c_^2^)/3
4361 reflections	(Δ/σ)_max_ = 0.001
158 parameters	Δρ_max_ = 1.15 e Å^−^^3^
0 restraints	Δρ_min_ = −1.49 e Å^−^^3^

### 2-Bromopyridinium dibromidoaurate(I)–2-bromopyridine (1/1) (5) Fractional atomic coordinates and isotropic or equivalent isotropic displacement parameters (Å^2^)

**Table:**

	*x*	*y*	*z*	*U*_iso_*/*U*_eq_	
Au1	0.40613 (2)	0.19800 (2)	0.14237 (2)	0.01475 (4)	
Br1	0.70783 (4)	0.20253 (4)	0.04441 (3)	0.01937 (7)	
Br2	0.10288 (4)	0.19977 (4)	0.24127 (3)	0.01916 (7)	
Br3	0.87521 (4)	0.80705 (4)	0.45816 (3)	0.01607 (7)	
Br4	0.61910 (4)	0.20705 (4)	0.37952 (3)	0.01548 (7)	
N11	0.7993 (3)	0.5993 (3)	0.3080 (2)	0.0148 (5)	
H11	0.777 (5)	0.537 (5)	0.372 (4)	0.023 (10)*	
C12	0.8486 (4)	0.7439 (4)	0.3104 (3)	0.0136 (6)	
C13	0.8796 (4)	0.8381 (4)	0.2066 (3)	0.0166 (6)	
H13	0.915444	0.939910	0.207838	0.020*	
C14	0.8574 (4)	0.7810 (4)	0.1010 (3)	0.0169 (6)	
H14	0.874796	0.845044	0.029191	0.020*	
C15	0.8098 (4)	0.6307 (4)	0.0997 (3)	0.0185 (7)	
H15	0.797788	0.589640	0.026693	0.022*	
C16	0.7804 (4)	0.5419 (4)	0.2048 (3)	0.0192 (7)	
H16	0.746428	0.439010	0.205056	0.023*	
N21	0.7050 (3)	0.4137 (3)	0.5260 (2)	0.0139 (5)	
C22	0.6507 (4)	0.2726 (4)	0.5288 (3)	0.0133 (6)	
C23	0.6153 (4)	0.1734 (4)	0.6295 (3)	0.0163 (6)	
H23	0.578118	0.072941	0.624963	0.020*	
C24	0.6360 (4)	0.2260 (4)	0.7371 (3)	0.0177 (6)	
H24	0.613599	0.161686	0.808677	0.021*	
C25	0.6900 (4)	0.3744 (4)	0.7390 (3)	0.0183 (6)	
H25	0.702604	0.414288	0.812244	0.022*	
C26	0.7251 (4)	0.4629 (4)	0.6319 (3)	0.0169 (6)	
H26	0.765117	0.562785	0.633051	0.020*	

### 2-Bromopyridinium dibromidoaurate(I)–2-bromopyridine (1/1) (5) Atomic displacement parameters (Å^2^)

**Table:**

	*U*^11^	*U*^22^	*U*^33^	*U*^12^	*U*^13^	*U*^23^
Au1	0.01907 (6)	0.01413 (7)	0.01280 (6)	−0.00329 (4)	−0.00677 (4)	0.00052 (4)
Br1	0.02015 (15)	0.02330 (17)	0.01494 (15)	−0.00386 (12)	−0.00459 (12)	−0.00038 (12)
Br2	0.01936 (15)	0.02198 (17)	0.01792 (16)	−0.00588 (12)	−0.00596 (12)	−0.00229 (12)
Br3	0.01851 (14)	0.01701 (16)	0.01330 (14)	−0.00387 (11)	−0.00431 (11)	−0.00125 (11)
Br4	0.01940 (14)	0.01518 (15)	0.01382 (15)	−0.00519 (11)	−0.00620 (11)	−0.00023 (11)
N11	0.0184 (12)	0.0133 (13)	0.0127 (13)	−0.0013 (10)	−0.0049 (10)	0.0030 (10)
C12	0.0130 (13)	0.0140 (15)	0.0132 (14)	−0.0003 (11)	−0.0035 (11)	−0.0005 (11)
C13	0.0175 (14)	0.0155 (16)	0.0162 (15)	−0.0063 (12)	−0.0014 (12)	−0.0001 (12)
C14	0.0177 (14)	0.0179 (16)	0.0150 (15)	−0.0039 (12)	−0.0043 (12)	0.0063 (12)
C15	0.0237 (16)	0.0185 (16)	0.0131 (15)	−0.0015 (13)	−0.0050 (12)	−0.0020 (12)
C16	0.0261 (16)	0.0146 (16)	0.0186 (16)	−0.0032 (13)	−0.0091 (13)	0.0007 (12)
N21	0.0193 (12)	0.0103 (12)	0.0121 (12)	−0.0014 (10)	−0.0047 (10)	0.0011 (9)
C22	0.0135 (13)	0.0134 (15)	0.0130 (14)	−0.0003 (11)	−0.0037 (11)	−0.0032 (11)
C23	0.0183 (14)	0.0126 (15)	0.0176 (16)	−0.0038 (12)	−0.0031 (12)	−0.0001 (12)
C24	0.0224 (15)	0.0154 (16)	0.0129 (15)	−0.0023 (12)	−0.0008 (12)	0.0013 (12)
C25	0.0241 (16)	0.0173 (16)	0.0135 (15)	−0.0004 (13)	−0.0061 (12)	−0.0007 (12)
C26	0.0239 (15)	0.0108 (15)	0.0164 (16)	−0.0007 (12)	−0.0069 (12)	−0.0014 (12)

### 2-Bromopyridinium dibromidoaurate(I)–2-bromopyridine (1/1) (5) Geometric parameters (Å, º)

**Table:**

Au1—Br1	2.3790 (4)	C15—H15	0.9500
Au1—Br2	2.3851 (4)	C16—H16	0.9500
Br3—C12	1.864 (3)	N21—C22	1.327 (4)
Br4—C22	1.904 (3)	N21—C26	1.352 (4)
N11—C12	1.345 (4)	C22—C23	1.380 (4)
N11—C16	1.346 (4)	C23—C24	1.382 (5)
N11—H11	0.87 (4)	C23—H23	0.9500
C12—C13	1.380 (4)	C24—C25	1.392 (5)
C13—C14	1.381 (5)	C24—H24	0.9500
C13—H13	0.9500	C25—C26	1.385 (5)
C14—C15	1.383 (5)	C25—H25	0.9500
C14—H14	0.9500	C26—H26	0.9500
C15—C16	1.368 (5)		
			
Br1—Au1—Br2	178.713 (12)	C15—C16—H16	119.8
C12—N11—C16	121.2 (3)	C22—N21—C26	116.2 (3)
C12—N11—H11	123 (3)	N21—C22—C23	125.6 (3)
C16—N11—H11	116 (3)	N21—C22—Br4	115.6 (2)
N11—C12—C13	120.5 (3)	C23—C22—Br4	118.8 (2)
N11—C12—Br3	116.9 (2)	C22—C23—C24	117.4 (3)
C13—C12—Br3	122.6 (2)	C22—C23—H23	121.3
C12—C13—C14	118.5 (3)	C24—C23—H23	121.3
C12—C13—H13	120.7	C23—C24—C25	119.1 (3)
C14—C13—H13	120.7	C23—C24—H24	120.5
C13—C14—C15	120.1 (3)	C25—C24—H24	120.5
C13—C14—H14	119.9	C26—C25—C24	118.6 (3)
C15—C14—H14	119.9	C26—C25—H25	120.7
C16—C15—C14	119.2 (3)	C24—C25—H25	120.7
C16—C15—H15	120.4	N21—C26—C25	123.1 (3)
C14—C15—H15	120.4	N21—C26—H26	118.4
N11—C16—C15	120.4 (3)	C25—C26—H26	118.4
N11—C16—H16	119.8		
			
C16—N11—C12—C13	0.6 (4)	C26—N21—C22—C23	−0.6 (4)
C16—N11—C12—Br3	−178.5 (2)	C26—N21—C22—Br4	178.9 (2)
N11—C12—C13—C14	0.5 (4)	N21—C22—C23—C24	0.9 (5)
Br3—C12—C13—C14	179.6 (2)	Br4—C22—C23—C24	−178.7 (2)
C12—C13—C14—C15	−1.8 (5)	C22—C23—C24—C25	0.3 (4)
C13—C14—C15—C16	1.9 (5)	C23—C24—C25—C26	−1.5 (5)
C12—N11—C16—C15	−0.6 (5)	C22—N21—C26—C25	−0.7 (4)
C14—C15—C16—N11	−0.7 (5)	C24—C25—C26—N21	1.7 (5)

### 2-Bromopyridinium dibromidoaurate(I)–2-bromopyridine (1/1) (5) Hydrogen-bond geometry (Å, º)

**Table:**

*D*—H···*A*	*D*—H	H···*A*	*D*···*A*	*D*—H···*A*
N11—H11···N21	0.87 (4)	1.99 (4)	2.861 (4)	174 (4)
C16—H16···Br1	0.95	2.88	3.675 (3)	142
C13—H13···Br2^i^	0.95	2.93	3.853 (3)	163
C26—H26···Br2^ii^	0.95	2.99	3.843 (3)	151
C26—H26···Br3	0.95	2.87	3.616 (3)	136
C16—H16···Br4	0.95	2.83	3.584 (3)	137

Symmetry codes: (i) *x*+1, *y*+1, *z*; (ii) −*x*+1, −*y*+1, −*z*+1.
